# Abiraterone acetate plus LHRH therapy versus abiraterone acetate while sparing LHRH therapy in patients with progressive, metastatic and chemotherapy-naïve, castration-resistant prostate cancer (SPARE): study protocol for a randomized controlled trial

**DOI:** 10.1186/s13063-017-2195-x

**Published:** 2017-10-04

**Authors:** Carsten-Henning Ohlmann, Michelle Jäschke, Peter Jaehnig, Susane Krege, Jürgen Gschwend, Heidrun Rexer, Michael Stöckle

**Affiliations:** 1 Department of Urology, Saarland University, Kirrbergerstrasse, 66421 Homburg/Saar, Germany; 2 PJ statistics, Berlin, Germany; 3 Department of Urology, Klinikum Essen Mitte, Essen, Germany; 4 Department of Urology, Klinikum Rechts der Isar, Technical University Munich, Munich, Germany; 5 Working Group on Urological Oncology (AUO), Germany Cancer Society, Berlin, Germany

**Keywords:** Castration-resistant prostate cancer, LHRH therapy, Abiraterone, Testosterone, Luteinizing hormone

## Abstract

**Background:**

The value of continuation of luteinizing hormone-releasing hormone (LHRH) therapy in castration-resistant prostate cancer (CRPC) remains controversial and clear evidence is lacking. Argumentation for cessation of LHRH therapy is the prolonged suppression of testosterone levels after the withdrawal of LHRH analogues and the fact that disease progression occurs despite castration levels of testosterone. Especially upon treatment with the life-prolonging cytochrome P450 17-alpha-hydroxylase (Cyp17)-inhibitor, abiraterone, which has the ability to further suppress testosterone serum levels over LHRH therapy alone, continuation of LHRH therapy seems to be negligible. However, the proven increase of luteinizing hormone levels after LHRH withdrawal, which is even further increased by abiraterone, may counteract the effects of abiraterone by the induction of enzymes of steroidogenesis. Therefore, cessation of LHRH therapy when starting treatment with abiraterone in CRPC may display an unpredictable hazard to the patients. This study will explore the role of continuation of LHRH therapy when starting treatment with abiraterone in patients with asymptomatic or mildly symptomatic, chemotherapy-naïve CPRC.

**Methods/design:**

The trial will assess radiographic progression-free survival after 12 months of treatment with abiraterone/prednisone in patients who will be randomized to receive continuing LHRH therapy versus LHRH withdrawal at the time of starting abiraterone therapy.

**Discussion:**

This multicenter, prospective, randomized, exploratory phase-II trial will bring about new data regarding the efficacy and safety of abiraterone/prednisone treatment with or without continuation of LHRH therapy. In addition, further insight into the complex hormonal changes under treatment will be gained and the results of this trial may give rise to a larger phase-III trial to examine the possibility of withdrawing LHRH therapy in patients with CRPC.

**Trial registration:**

ClinicalTrials.gov, ID: NCT02077634. Registered on 9 December 2013.

**Electronic supplementary material:**

The online version of this article (doi:10.1186/s13063-017-2195-x) contains supplementary material, which is available to authorized users.

## Background

Treatment of patients with progressive, castration-resistant prostate cancer (CRPC) with abiraterone in combination with prednisone leads to an increase in progression-free and overall survival pre- and post docetaxel as evidenced in two large, phase-III trials (COU-AA-301 [1], COU-AA-302 [2, 3]). Inclusion of patients in both phase-III and former trials required continuation of medical castration with luteinizing hormone-releasing hormone (LHRH) therapy in patients who had not undergone prior surgical castration. Therefore, the label on abiraterone (Zytiga®) contains advice to continue LHRH therapy during treatment with it [4]. However, in medically castrated men treated with abiraterone while continuing on LHRH therapy, testosterone further decreased rapidly to undetectable levels [5]. This potential of abiraterone to achieve even a more complete androgen deprivation than LHRH therapy alone appeals to speculation as to whether abiraterone may achieve and maintain testosterone deprivation at undetectable levels without concomitant LHRH therapy and that LHRH therapy may be discontinued at the initiation of therapy with abiraterone.

In general, cessation of LHRH therapy in patients with castration-resistant prostate cancer is studied insufficiently and, therefore, current guidelines advise to continue medical castration [6]. In contrast, there is little evidence from previous clinical trials that continuation of castration leads to only a marginal, if any, survival benefit [7, 8].

This aside, even if abiraterone maintains androgen deprivation in the absence of LHRH therapy, treatment without concomitant LHRH therapy may impair the efficacy of abiraterone due to the effects of LHRH therapy independent of androgen deprivation. Cessation of LHRH therapy leads to rapid recovery of luteinizing hormone (LH) levels after a median of 58 days to 4.5 months, even after long-term medical castration [9, 10]. The rise in LH levels is further enhanced since treatment with abiraterone in non-castrated mice induces a sustained increase in LH levels despite suppression of testosterone [11]. This feedback mechanism was confirmed in non-castrated patients since treatment with abiraterone can lead to an increase in LH levels that return to normal after cessation of therapy [12]. Apart from its action on the testes and adrenal glands, LH may, at least in part, act directly on prostate cancer cells via LH-specific receptors causing an increase in the expression of several key steroidogenic enzymes including steroidogenic acute regulatory protein (StAR), CYB5B, CYP11A and 3-beta-hydroxysteroid dehydrogenase (3βHSD) [13]. Induction of these enzymes has recently been shown in xenograft models with primary or secondary resistance against abiraterone and was deemed one possible cause of resistance [14]. The activation of these enzymes may enhance rescue pathways of testosterone and dihydrotestosterone (DHT) production in prostate cancer cells [15] and thereby impair the efficacy of abiraterone.

Taken together, discontinuation of LHRH therapy during treatment with abiraterone may impair the efficacy of abiraterone and display an unpredictable hazard to patients leading to early progression, and consequently shortened cancer-specific and overall survival.

Investigation of the effects of discontinuing LHRH therapy at the time of initiation of abiraterone treatment therefore represents a major medical need, especially in view of the side effects of LHRH therapy. This will be the first study to assess the efficacy of abiraterone acetate in patients with CRPC randomized to continue or discontinue LHRH therapy. The results of this trial will contribute evidence to the understanding of castration- and hormone-resistant prostate cancer. Furthermore, this trial may add evidence to the current recommendation to continue LHRH therapy, thereby improving the benefit of abiraterone treatment for the patient. In order to enable comparison of the trial results to previous trials, the main aspects of the trial design are in close similarity to those of the COU-AA-302 trial protocol [2].

## Methods/design

### Hypothesis

**Fig. 1:**
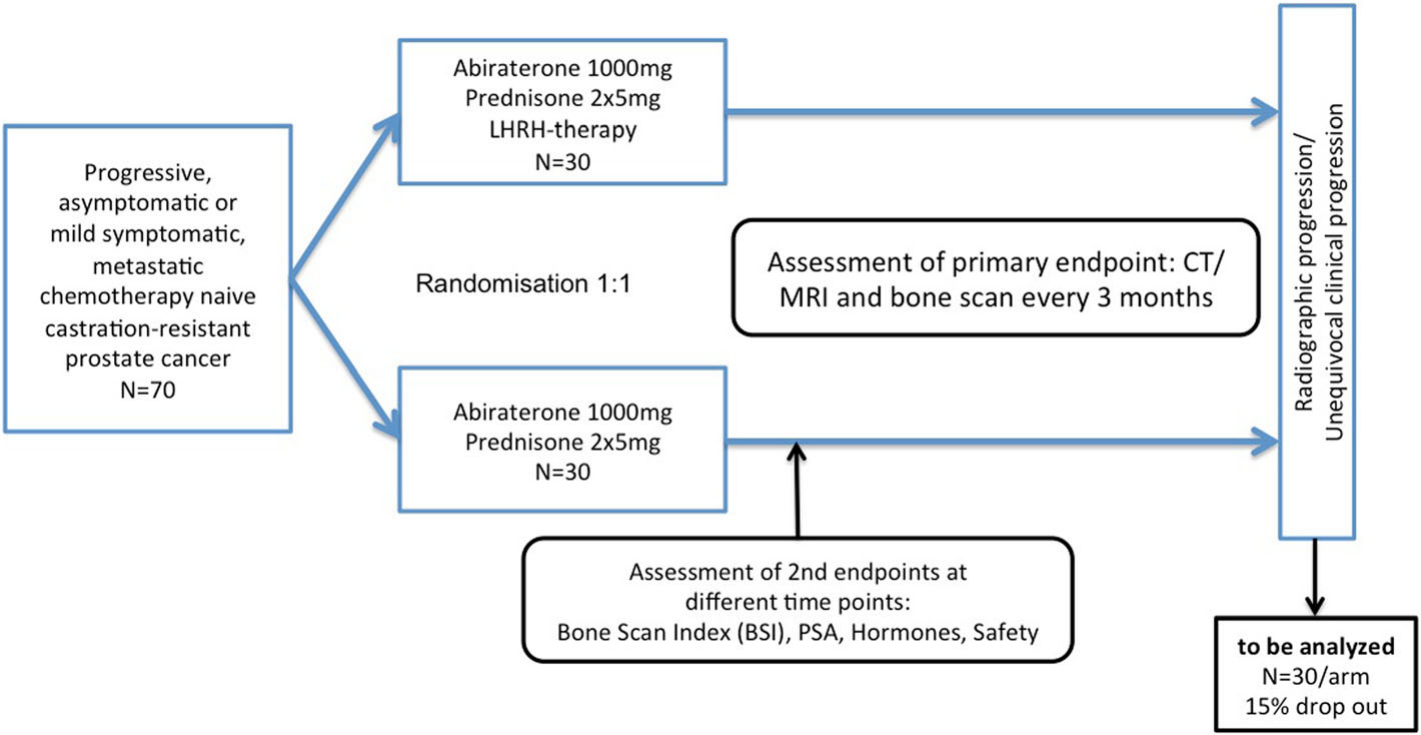
Schematic trial design from screening to final analysis. Patients will be randomized to receive abiraterone/prednisone ± continuation of LHRH therapy until radiographic progression or unequivocal clinical progression. Assessment of progression by computed tomography (CT)/magnetic resonance imaging (MRI) and bone scan will be repeated every 3 months. Bone Scan Index (BSI) will be analyzed at baseline and after 3 and 6 months. Hormone analysis includes FSH, LH, LHRH, DHEA, testosterone and dihydrotestosterone that will be measured at baseline, at months 1, 2 and 3, and every 3 months thereafter

Given the risk that discontinuation of LHRH therapy patients, leading to early progression and consequent shortening of cancer-specific and overall survival between patients, leading to early progression and consequent shortening of cancer-specific and overall survival between study arms, this phase II study was designed. This is a two-arm, investigator-initiated, randomized controlled trial (RCT) that compares the effect of continuous LHRH therapy compared to cessation of LHRH therapy when commencing abiraterone in patients with castration-resistant, chemotherapy-naïve prostate cancer (NCT02077634). The trial has been developed in accordance with Standard Protocol Items: Recommendations for Interventional Trials (SPIRIT) guidelines (Additional files 1 and 2) and the study design is described in Fig. 1.

### Randomization

Patients who meet the inclusion and exclusion criteria and gave written informed consent will be randomized through an electronic Case Report Form (eCRF). All trial sites must register at the website of the trial specific system. When an investigator has chosen a certain patient, the basic information on the patient have to be documented before the patient will be randomly assigned in a 1:1 ratio to one of the treatment arms. Randomization will follow a block randomization with a block size of 6, ensuring that after each block the number of patients in both treatment arms is equal. Randomized treatment will be confirmed by e-mail send to the investigator.

### Interventions and trial flow

Depending on the treatment arm, patients will receive the following treatments until confirmed radiographic progression, unequivocal clinical progression or unacceptable toxicity occurs (see Additional file 1) (Table 1):

**Table 1:** Scheduled events

		Treatment phase (cycle length = 28 days)							Follow-up phase
Required investigations	Baseline day −28 to 0	Cycle 1 Day 1	Cycle 1 Day 15	Cycle 2 and 3 Day 1	Cycles 4, 7, 10 (continue every 3rd cycle) Day 1	Any other cycle, Day 1	At treatment discontinuation	End-of-treatment visit	Follow-up visit (every 3 months)
Procedures									
Signed consent form	X								
Medical history, prior Prostate cancer therapies	X								
Worst pain within last 24 h	X	X	X	X	X	X		X	
Physical exam and weight	X		X	X	X	X		X	
Vital signs	X	X	X	X	X	X		X	
ECOG	X	X	X	X	X	X		X	
12-lead ECG	X				X			X	
Cardiac echo	X				X				
Dosing compliance			X	X	X	X		X	
Concomitant medication	X	X	X	X	X	X		X	
Adverse events	X	X	X	X	X	X		X	X
Laboratory assessments									
Hematology	X			X	X	X		X	
Serum chemistry, electrolytes	X		X	X	X	X		X	
Liver function test	X		X	X	X	X		X	
Serum lipids	X		X	X	X				
Plasma glucose	X		X	X	X	X		X	
PSA		X		X	X	X		X	X
Serum testosterone, hormones		X		X	X		X	X	
Tumor assessments									
CT/MRI	X				X			X	X
Bone scan	X				X			X	X
Disease progression assessments					X		X	X	X
Next therapy for prostate cancer									X

*CT* computed tomography, *ECG* electrocardiogram, *ECOG*, Eastern Cooperative Oncology Group, *MRI* magnetic resonance imaging, *PSA* prostate-specific antigen

#### Abiraterone acetate

Patients will be instructed to take four tables (abiraterone acetate) per os at least 1 h before a meal or 2 h after a meal any time up to 10 p.m. every day.

#### Prednisone

Patients will be instructed to take 5-mg prednisone or prednisolone tablets per os, twice daily.

#### LHRH therapy

Patients who are randomized to the AA + Pred + LHRH therapy arm (arm A) will continue on the same LHRH therapy that had been used prior to randomization. LHRH therapy (LHRH analogue or LHRH antagonist) will be administered according to the labeling information of the specific drug.

### Criteria for discontinuation of study treatment

Criteria for discontinuation are based on the COU-AA-302 trial protocol [2]. Patients should ordinarily be maintained on study treatment until confirmed radiographic progression. If the patient has radiographic progression but no unequivocal clinical progression and alternate treatment is not initiated, the patient may continue on study treatment at the investigator’s discretion. However, if patients have unequivocal clinical progression without radiographic progression, these patients are indicated for the current standard of care. Study treatment should be stopped and patients advised regarding available treatment options.

Similar to the COU-AA-302 trial [2], unequivocal clinical progression will be characterized for this study as:

1. Cancer pain requiring initiation of chronic administration of opiate analgesia (oral opiate use for ≥ 3 weeks; parenteral opiate use for ≥ 7 days
*OR*
2. Immediate need to initiate cytotoxic chemotherapy or the immediate need to have either radiation therapy or surgical intervention for complications due to tumor progression, even in the absence of radiographic evidence of disease progression
*OR*
3. Deterioration in Eastern Cooperative Oncology Group (ECOG) performance status to grade 3 or higher.
  Patients whose ECOG performance status decreases to grade 2 during the study should be assessed carefully for their need for docetaxel therapy

When study treatment is discontinued due to unequivocal clinical progression, the investigator should obtain further imaging studies to assess for radiographic progression, including a confirmatory bone scan, as appropriate.

Study treatment will be continued on patients who have increasing prostate-specific antigen (PSA) values in the absence of radiographic or unequivocal clinical progression. Although serial PSAs will be measured on this study, progression or change in PSA values is not considered a reliable measure of disease progression, and should not be used as an indication to discontinue study therapy.

At the time of discontinuation of study treatment, re-initiation of LHRH therapy is mandatory and must be recorded in patients who were randomized to the abiraterone plus prednisone arm.

### Study objectives

The primary objective of the study is to analyze the clinical benefit of abiraterone acetate plus prednisone while sparing LHRH therapy in chemotherapy-naïve patients with metastatic, castration-resistant prostate cancer (CRPC). The secondary objectives of this study include correlation of early PSA response to treatment with abiraterone with radiographic progression-free survival (rPFS), evaluation of the Bone Scan Index (BSI) in the early course of treatment as a biomarker for response to treatment, changes on hormones of the pituitary-gonadal axis, and characterization of the safety profile of abiraterone acetate while sparing LHRH therapy in comparison to continuing LHRH therapy.

### Study endpoints

#### Primary efficacy endpoint

The primary endpoint of the study is rate of rPFS at month 12 based on parameters suggested by PCWG2 [16] and modified Response Evaluation Criteria in Solid Tumors (RECIST) as the time from randomization to the occurrence of one of the following:

1. A patient is considered to have progressed by bone scan if:
  - The first bone scan with at least two new lesions compared to baseline is observed less than 12 weeks from randomization and is confirmed by a second bone scan taken at least 6 weeks later showing at least two additional new lesions (a total of at least four lesions compared to baseline)
  - The first bone scan with at least two new lesions compared to baseline is observed at least 12 weeks from randomization and the new lesions are verified on the next bone scan at least 6 weeks later (a total of at least two new lesions compared to baseline).
2. Progression of soft tissue lesions measured by computed tomography (CT) or magnetic resonance imaging (MRI) as defined by modified RECIST criteria
3. Death from any cause

In the analysis of rPFS, the following censoring rules apply:

1. If the patient does not a have baseline scan or on-study scans, the patient will be censored on the date of randomization
2. If the patient does not show progression according to modified RECIST, the patient will be censored on the date of the last scheduled scan
3. Bone scan censoring rules
  - if the patient remains on study treatment and prior scans do not show radiographic progression, the patient will be censored on the date of the last scan showing no disease progression
  - if the patient discontinues study treatment for any reason and progression was not observed in the scans prior to discontinuation, the patient will be censored on the last scan showing no disease progression
  - if the patient discontinues study treatment for any reason and additional new lesions were observed in the scan prior to the discontinuation, and there was no confirmatory scan, the patient will be censored on the date of the last scan that showed no disease progression
4. Patients will also be censored on the date of the last scan that shows no disease progression if:
  - the patient receives another therapy known or intended for treatment of metastatic CRPC during the study
  - the patient misses at least two planned radiographic scans or has at least two consecutive unreadable scans
  - the patient has unequivocal progression of non-bone, non-target lesions (e.g., appearance of non-measurable visceral metastases or pathologically confirmed malignant effusions)

#### Secondary efficacy endpoints

- PSA response rate scored in patients achieving a post-treatment PSA decline of at least 50% according to the protocol-specific PCWG2 criteria
- Time to PSA progression will be measured from the time interval from the date of randomization to the date of the PSA progression as defined in the protocol-specific PCWG2 criteria. The determination of PSA progression will require that the patient receive at least three cycles of therapy
- Objective response rate in patients with measurable disease (RECIST)
- Value of the BSI as a biomarker of response to treatment
- Changes in pituitary gonadal axis by measurement of androgens and hormones (LHRH, LH, FSH, testosterone, DHT)
- Safety

### Main inclusion criteria (based on the COU-AA-302 trial protocol [2])

1. Male aged 18 years and above
2. Histologically or cytologically confirmed adenocarcinoma of the prostate
3. Metastatic disease documented by positive CT/MRI and/or bone scan. If lymph node metastasis is the only evidence of metastasis, it must be ≥ 2 cm in diameter
4. Prostate cancer progression documented by PSA according to PCWG2 or radiographic progression according to modified RECIST criteria
5. Asymptomatic or mildly symptomatic from prostate cancer. A score of 0–1 for the question of worst pain within the last 24 h will be considered asymptomatic, and a score of 2–3 will be considered mildly symptomatic
6. Medically castrated, with testosterone levels of < 20–50 ng/dl (<2.0 nM)
7. Combined androgen blockade is permitted, but not required. If patients have received combined androgen blockade with an anti-androgen they must have shown PSA progression after discontinuing the anti-androgen prior to enrollment (at least 4 weeks since last flutamide, at least 6 weeks since last bicalutamide or nilutamide)

### Main exclusion criteria (based on the COU-AA-302 trial protocol [2])

1. Surgical castration (i.e., orchiectomy)
2. Application of any LHRH therapy (LHRH analogue or LHRH antagonist) within 3 months (for patients receiving a 3-month formulation) or 1 month (for patients receiving a 1-month formulation) prior to cycle 1, day 1
3. Patients receiving a 6- or 12-month formulation of LHRH therapy
4. Active infection or other medical condition that would make prednisone/prednisolone (corticosteroid) use contraindicated
5. Any chronic medical condition requiring a higher dose of corticosteroid than 5 mg prednisone/prednisolone twice daily
6. Pathological finding consistent with small-cell carcinoma of the prostate
7. Liver or visceral organ metastasis
8. Known brain metastasis
9. Use of opiate analgesics for cancer-related pain, including codeine, tramadol, tilidin and others, currently or anytime within 4 weeks of cycle 1, day 1
10. Prior cytotoxic chemotherapy or biological therapy for the treatment of CRPC
11. Radiation therapy for treatment of the primary tumor within 6 weeks of cycle 1, day 1
12. Radiation or radionuclide therapy for treatment of metastatic CRPC
13. Prior treatment with abiraterone acetate or other CYP17 inhibitors (ketoconazole, TAK700, TOK001), enzalutamide (Xtandi) or investigational agents targeting the androgen receptor for prostate cancer for more than 7 days
14. Prior systemic treatment with an azole drug (e.g., fluconazole, itraconazole) within 4 weeks of cycle 1, day 1
15. Prior flutamide (Eulexin) treatment within 4 weeks of cycle 1, day 1 (patients whose PSA did not decline for three or more months in response to an anti-androgen given as a second-line or later intervention will require only a 2-week washout prior to cycle 1, day 1)
16. Bicalutamide (Casodex), nilutamide (Nilandron) within 6 weeks of cycle 1, day 1 (patients whose PSA did not decline for three or more months in response to an anti-androgen given as a second-line or later intervention will require only a 2-week washout prior to cycle 1, day 1)
17. Uncontrolled hypertension (systolic BP ≥ 160 mmHg or diastolic BP ≥ 95 mmHg). Patients with a history of hypertension are allowed provided that blood pressure is controlled by anti-hypertensive treatment
18. Active or symptomatic viral hepatitis or chronic liver disease
19. History of pituitary or adrenal dysfunction
20. Clinically significant heart disease as evidenced by myocardial infarction, or arterial thrombotic events in the past 6 months, severe or unstable angina, or New York Heart Association (NYHA) class II–IV heart disease or cardiac ejection fraction measurement of < 50% at baseline

## Statistics

Primary analysis for radiographic progression-free survival (rPFS) will be based on the full analysis set. For this trial, the primary efficacy parameter will be the rate of rPFS after 12 months. No statistical comparison is planned between the two treatment arms. However, with a one-sided 10% type-I error and power of 80%, at least 28 patients in arm B (AA + prednisolone) need to be randomized in order to test the following hypotheses:

- H0 (null): a rPFS rate at 12 months of 60% (uninteresting to pursue any further investigation)
- H1 (alternative): a rPFS rate at 12 month of 40% (warrants further investigation in a phase-III trial)

The hypotheses regarding an anticipated rPFS rate at 12 months of 40% and an of no interest rate of 60% are based on the observed rPFS in the control and treatment arms of the COU-AA-302 trial [2]. The control arm in the SPARE trial (arm A) will serve as a calibration for the analysis of the hormones of the pituitary-gonadal axis and reduction of a selection bias.

Assuming a dropout rate of 15% in each arm it is estimated that 70 patients need to be recruited for this trial at 12 sites across Germany. Accrual time for the trial is estimated to take 12 months.

### Statistical analysis

Prior to locking the database, a data review is planned in order to review individual data and validate the Statistical Analysis Plan. All deviations from protocol definitions will be listed and defined as major or minor deviations.

Statistical analyses will be performed using eCRF data collected until a clinical cut-off date is reached that is defined when the number of events required for the final analysis of the efficacy variables is achieved. Unless otherwise specified, all continuous endpoints will be summarized using descriptive statistics, which will include the number of patients (*n*), mean, standard deviation, median, minimum, and maximum. All categorical endpoints will be summarized using frequencies and percentages. The baseline measurement will be the last value on, or before, the date of first study treatment. Unless otherwise stated, the calculation of proportions will based on the sample size of the population of interest. Kaplan-Meier curves will be used to describe event-free rates over time. Median event-free times by treatment arm will be reported with 95% CI, if the number of events allows the estimation of the median. The rPFS rate at 12 month will be estimated by the Kaplan-Meier method. These rates estimate the proportion of patients who do not progress and are alive at a given time. The 95% CI for the rPFS rate will be estimated using Greenwood’s estimate of the standard error (SE) and a linear transformation of the PFS function.

## Definition of analysis population

Patient disposition and efficacy analyses will be performed on data from the intention-to-treat (ITT) population. All patients randomized into the study will be classified according to their assigned treatment group, regardless of the actual treatment received. The primary efficacy analyses will be on the ITT basis. All patients who receive any part of abiraterone acetate will be included in the analysis of safety (safety population).

## Risk-benefit assessment

Patients randomized to arm A (AA + Pred + LHRH therapy) will receive the approved standard treatment for this indication. Therefore, the risks of the therapy may not be distinguished from the risks of therapy the patient would receive if not participating in the trial.

Patients randomized to arm B (AA + Pred) will receive less treatment compared to the approved standard treatment for this indication. The risks for the patients are only based on the effect of sparing LHRH therapy on the primary endpoint of the trial, i.e., radiographic PFS. If the hypothesis of the trial holds true, patients in arm B may experience radiographic progression earlier compared to patients randomized to arm A. Shortening of time to radiographic progression cannot not be predicted from the available results of studies conducted with abiraterone acetate. However, patients who experience radiographic progression are able to receive further treatment for advanced prostate cancer since several therapies have been approved for this indication (i.e., docetaxel, cabazitaxel, radium-223). Therefore, the risk on overall survival for patients participating in this trial is considered to be minimal. In contrast, patients randomized to arm B may benefit from sparing LHRH therapy and, therefore, do not experience side effects from treatment related to the application of LHRH therapy or its toxicities.

## Discussion

There is still a debate as to whether continuation of LHRH therapy in patients with CRPC is mandatory due to lack of evidence [17]. Until otherwise proven, guidelines recommend continuing on LHRH therapy [6]. However, continuation of LHRH therapy may cause considerable side effects and unnecessary expenses [18].

Especially in view of the current available treatment options for CRPC including taxanes (docetaxel, cabacitaxel), CYP17 inhibitors (abiraterone) and androgen-receptor inhibitors (enzalutamide), continuation of LHRH therapy may depend on the actual treatment given. With regard to serum testosterone levels, there is plenty of evidence that levels remain low after long-term treatment with LHRH agonists [9, 10, 19]. With this regard, cessation of LHRH therapy may have only little clinical relevance since serum testosterone levels remain low and may not affect proliferation of cancer cells. However, in contrast to serum testosterone levels, recovery of serum LH levels may occur after several weeks to months after cessation of LHRH therapy [9, 10, 19]. This rise in LH levels, which is even increased by abiraterone itself [12], and subsequent induction of the expression of several enzymes of steroidogenesis may counteract the proposed inhibition of intracellular androgen signaling by abiraterone. Currently, there are several clinical trials underway investigating the efficacy of abiraterone in different clinical scenarios (summarized in [17]). However, in any of these trials abiraterone will be combined with a backbone of LHRH therapy. The NCT02077634 (SPARE) trial is, therefore, currently the only trial that will give further insight into the complex hormonal changes under treatment with abiraterone with or without concurrent LHRH therapy. In case of an rPFS rate of ≤ 40% or less at month 12 in arm B (abi + Pred), further evaluation as to whether LHRH therapy has to be continued upon treatment with abiraterone in a phase-III trial is warranted. The results of the most recently published LATITUDE trial [20], showing that the addition of abiraterone to LHRH therapy in castration-sensitive prostate cancer (CSPC) leads to significant survival benefit over LHRH therapy alone, further increases the medical need to evaluate the efficacy of abiraterone treatment while sparing LHRH therapy. Becoming a new standard treatment for CSPC, the number of patients receiving the combination of AA + Pred + LHRH therapy will increase, not knowing whether LHRH therapy is necessary.

## Trial status

Recruitment for the trial started in May 2014 and is expected to end in May 2017.

## Additional files

Additional file 1: Trial protocol. (PDF 1322 kb)

Additional file 2: SPIRIT 2013 Checklist: recommended items to address in a clinical trial protocol and related documents*. (PDF 101 kb)

## Abbreviations

3βHSD: 3-beta-hydroxysteroid dehydrogenase
AA: Abiraterone dcetate
BP: Blood pressure
BSI: Bone Scan Index
CRPC: Castration-resistant prostate cancer
CSPC: Castration-sensitive prostate cancer
CT: Computed tomography
CYB5B: Cytochrome B5b
CYP11A: Cytochrome P450-11A
CYP17: Cytochrome P450 17-alpha-hydroxylase
DHT: Dihydrotestosterone
FSH: Follicle-stimulating hormone
IIT: Intention-to-treat
LH: Luteinizing hormone
LHRH: Luteinizing hormone-releasing hormone
MRI: Magnetic Resonance Imaging
NYHA: New York Heart Association
PCWG2: Prostate Cancer Working Group
Pred: Prednisone
PSA: Prostate-specific antigen
RCT: Randomized controlled trial
RECIST: Response Evaluation Criteria in Solid Tumors
rPFS: Radiographic progression-free survival
StAR: Steroidogenic acute egulatory protein
