# Supplementary material for: Abiraterone acetate plus LHRH therapy versus abiraterone acetate while sparing LHRH therapy in patients with progressive, metastatic and chemotherapy-naïve, castration-resistant prostate cancer (SPARE): study protocol for a randomized controlled trial
Source: Trials. 2017 Oct 4;18:457. doi: 10.1186/s13063-017-2195-x (PMC5628432; doi:10.1186/s13063-017-2195-x)
Supplement: Supplementary file 1 — Trial protocol. (PDF 1322 kb) [file 13063_2017_2195_MOESM1_ESM.pdf]

Randomized phase-II trial of abiraterone acetate plus LHRH-therapy versus abiraterone acetate sparing LHRH-therapy in patients with progressive chemotherapy-naïve castration-resistant prostate cancer (SPARE)

Investigator-initiated Study (IIS)

SPARE-001/AP 67/11

German Clinical Trials Register (DRKS): DRKS00005987

Sponsor Universität des Saarlandes
Postfach 151150
66041 Saarbrücken

Version 2.0 of 30.04.2015

This study is financially supported by Janssen-Cilag GmbH, Neuss.

Contact addresses

Trial Coordinator/ Medical Monitor

Carsten-Henning Ohlmann, M.D.
Klinik für Urologie und Kinderurologie
Universitätsklinikum des Saarlandes
Kirrbergerstr. 1, 66421 Homburg
Germany

Phone: +49-6841-16-24700
Fax: +49-6841-16-24026
e-mail: carsten.ohlmann@uks.eu

CRO

Meckevidence
Seestr. 11
17252 Schwarz
Phone: +49-39827-79677
Fax: +49-39827-79678
e-mail: heidrun.rexer@meckevidence.de

Biostatistician

Pj statistics
Dipl.-Kfm. Peter Jähnig
Niedstr.16
12159 Berlin

Phone: +49 30 6005 6868
Mobile: +49 176 5716 4339
e-mail: peter.jaehnig@pj-statistics.com

Central Lab

Klinische Chemie und Laboratoriumsmedizin
Universitätsklinikum d. Saarlandes
Kirrbergerstr. 1, 66421 Homburg
Germany

Phone: +49-6841-16-30700
Fax: +49-6841-16-30703

Synopsis

Study Title:
Randomized phase-II trial of abiraterone acetate plus LHRH-therapy versus abiraterone acetate sparing LHRH-therapy in patients with progressive chemotherapy-naïve castration-resistant prostate cancer (SPARE)

Study Number:
SPARE-001 (AP 67/11)

Study Phase: 2

Primary Objective:
- The primary objective of the study is to analyze the clinical benefit of abiraterone acetate plus prednisone while sparing LHRH-therapy in chemotherapy-naïve patients with metastatic castration-resistant prostate cancer (CRPC).

Secondary Objectives:
- To establish additional clinically relevant information regarding early PSA responses to abiraterone and to correlate these with radiographic-progression free survival
- To investigate effects of both treatment arms on hormones of the pituitary gonadal axis
- To characterize the safety profile of abiraterone acetate while sparing LHRH-therapy in comparison to continuing LHRH-therapy

Study Design:
This is an exploratory Phase 2 multicenter, randomized, open-label study with a randomization allocation ratio of 1:1 [abiraterone acetate + prednisone + LHRH-therapy (Arm A) versus abiraterone acetate + prednisone (Arm B)]. For both groups patients will receive a dose of 1000 mg abiraterone acetate and 10mg prednisone daily (QD). Study drug will be administered as 4 x 250-mg abiraterone acetate tablets and prednisone will be administered as 5 mg orally twice a day (BID). Patients randomized to the LHRH-therapy group will receive the same LHRH-therapy they received prior to entering the trial

Study Population:
70 medically castrated male patients with metastatic CRPC who have shown tumor progression and are non- or mildly-symptomatic will be enrolled from approximately 12 German study sites.

Inclusion criteria
1. Willing and able to provide written informed consent
2. Written Data Protection Consent has been obtained
3. Male aged 18 years and above
4. Histologically or cytologically confirmed adenocarcinoma of the prostate
5. Metastatic disease documented by positive CT/MRI and/or bone scan (both must be performed).. If lymph node metastasis is the only evidence of metastasis, it must be ≥2 cm in diameter
6. Prostate cancer progression documented by PSA according to PCWG2 or radiographic progression according to modified RECIST criteria
7. Asymptomatic or mildly symptomatic from prostate cancer. A score of 0-1 for the question of worst pain within last 24 hours (Appendix 8) will be considered asymptomatic, and a score of 2-3 will be considered mildly symptomatic.
8. Medically castrated, with testosterone levels of <20-50 ng/dl (< 2.0 nM).
9. Combined androgen blockade is permitted, but not required. If patients received combined androgen blockade with an anti-androgen they must have shown PSA progression after discontinuing the anti-androgen prior to enrollment (≥4 weeks since last flutamide, ≥6 weeks since last bicalutamide or nilutamide).

10. Eastern Cooperative Oncology Group (ECOG) Performance Status of ≤2 (Appendix 6)
11. Hemoglobin ≥9.0 g/dL independent of transfusion
12. Platelet count ≥100,000 /µl
13. Serum albumin ≥3.0 g/dl
14. Serum creatinine < 1.5 x ULN or a calculated creatinine clearance ≥60 ml/min (Appendix 7)
15. Serum potassium ≥3.5 mmol/l
16. Liver function:
    a. Serum bilirubin <1.5 x ULN (except for patients with documented Gilbert's disease)
    b. AST or ALT <2.5 x ULN
17. Able to swallow the study drug whole as a tablet
18. Life expectancy of at least 6 months
19. Patients who have partners of childbearing potential must be willing to use a method of birth control with adequate barrier protection as determined to be acceptable by the principal investigator and sponsor during the study and for 1 week after last study drug administration.

Exclusion criteria

1. Surgical castration (i.e. orchiectomy).
2. Application of any LHRH-therapy (LHRH-analogue or LHRH-antagonist) within 3 months (for patients receiving a 3-months formulation) or 1 months (for patients receiving a 1-month formulation) prior to Cycle 1 day 1.
3. Patients receiving a 6- or 12-months formulation of LHRH-therapy
4. Active infection or other medical condition that would make prednisone/prednisolone (corticosteroid) use contraindicated
5. Any chronic medical condition requiring a higher dose of corticosteroid than 5mg prednisone/prednisolone bid.
6. Pathological finding consistent with small cell carcinoma of the prostate
7. Liver or visceral organ metastasis
8. Known brain metastasis
9. Use of opiate analgesics for cancer-related pain, including codeine, tramadol, tilidin and others (see Appendix 9), currently or anytime within 4 weeks of Cycle 1 Day 1.
10. Prior cytotoxic chemotherapy or biologic therapy for the treatment of CRPC
11. Radiation therapy for treatment of the primary tumour within 6 weeks of Cycle 1, Day 1
12. Radiation or radionuclide therapy for treatment of metastatic CRPC
13. Prior treatment with Abiraterone acetate or other CYP17 inhibitors (ketoconazole, TAK700, TOK001), Enzalutamide (Xtandi) or investigational agents targeting the androgen receptor for prostate cancer for more than 7 days
14. Prior systemic treatment with an azole drug (e.g. fluconazole, itraconazole) within 4 weeks of Cycle 1, Day 1
15. Prior flutamide (Eulexin) treatment within 4 weeks of Cycle 1, Day 1 (patients whose PSA did not decline for three or more months in response to antiandrogen given as a second line or later intervention will require only a two week washout prior to Cycle 1, Day 1)
16. Bicalutamide (Casodex), nilutamide (Nilandron) within 6 weeks of Cycle 1 Day 1 (patients whose PSA did not decline for three or more months in response to antiandrogen given as a second line or later intervention will require only a two week washout prior to Cycle 1, Day1)
17. Uncontrolled hypertension (systolic BP ≥160 mmHg or diastolic BP ≥95 mmHg). Patients with a history of hypertension are allowed provided that blood pressure is controlled by anti-hypertensive treatment
18. Active or symptomatic viral hepatitis or chronic liver disease
19. History of pituitary or adrenal dysfunction

20. Clinically significant heart disease as evidenced by myocardial infarction, or arterial thrombotic events in the past 6 months, severe or unstable angina, or New York Heart Association (NYHA) Class II-IV heart disease or cardiac ejection fraction measurement of <50 % at baseline
21. Any condition that requires treatment with Digoxin, digitoxin, and other digitalis drugs
22. Atrial Fibrillation, or other cardiac arrhythmia requiring therapy
23. Other malignancy with a ≥30 % probability of recurrence within 24 months, except non-melanoma skin cancer.
24. Administration of an investigational therapy within 30 days of Cycle 1, Day 1
25. Any condition, which, in the opinion of the investigator, would preclude participation in this trial.

Duration of Treatment:

Patients will have a screening period of up to 28 days prior to randomization on Cycle 1 Day 1. Each cycle consists of 28 days. Patients will be treated until disease progression as defined in the protocol. After discontinuing study treatment, patients will be contacted once every three months. Follow-up for each patient will continue until radiographic progression occurs, patient dies, is lost to follow-up, withdraws informed consent or until end of the study, whichever occurs earlier. Follow-up for all patients ends when rPFS at month 12 had been determined for the last patient on study treatment.

Efficacy Assessment:

The primary efficacy endpoint is the rate of radiographic progression-free survival (rPFS) at month 12

- Efficacy assessment in rPFS will utilize sequential imaging studies as defined by PCWG2 and modified RECIST criteria.

Secondary efficacy assessments:

- PSA response rate scored in patients achieving a post-treatment PSA decline of at least 50% according to the protocol-specific PCWG2 criteria
- Time to PSA-progression will be measured from the time interval from the date of randomization to the date of the PSA progression as defined in the protocol-specific PCWG2 criteria. The determination of PSA progression will require that the patient receive at least 3 cycles of therapy.
- Objective response rate in patients with measurable disease (RECIST)
- Value of the bone-scan index as a biomarker of response to treatment
- Changes in pituitary gonadal axis by measurement of androgens and hormones (LHRH, LH, FSH, testosterone, DHT)

Safety Assessments:

- Medical history, vital sign measurements, physical examination, and body weight Concomitant therapy and procedures
- Adverse events (AEs) and serious adverse events (SAEs), including laboratory test AEs will be graded and summarized according to the National Cancer Institute (NCI) Common Terminology Criteria for Adverse Events (CTCAE), version 3.0
- Blood chemistry, hematology, coagulation studies, serum lipids

Risk/benefit Analysis

<u>Arm A (abiraterone acetate + prednisone + LHRH-therapy)</u>

Patients randomized to Arm A will receive the approved standard treatment for this indication. Therefore the risks of the therapy may not be distinguished from the risks of therapy the patient would receive if not participating in the trial.

Arm B (abiraterone acetate + prednisone)
Patients randomized to Arm B will receive less treatment compared to the approved standard treatment for this indication. The risks for the patients are only based on the effect of sparing LHRH-therapy on the primary endpoint of the trial, i.e. radiographic progression free survival. If the hypothesis of the trial holds true, patients in Arm B may experience radiographic progression earlier compared to patients randomized to Arm A. Shortening of time to radiographic progression cannot not be predicted from the available results of studies conducted with abiraterone acetate. However, patients who experience radiographic progression are able to receive further treatment for advanced prostate cancer since several therapies have been approved for this indication (i.e. docetaxel, cabazitaxel, alpharadin). Therefore the risk on overall survival for patients participating in this trial is considered to be minimal.
In contrast, patients randomized to Arm B may benefit from sparing LHRH-therapy who therefore do not experience side effects from treatment related to the application of LHRH-therapy or toxicities.

Risks for both treatment arms
Risks that have to be taken into account for both treatment arms are radiographic assessments for the primary efficacy endpoint and drawing of blood samples for hormone analysis.
The Frequency of assessments of the primary study endpoint (i.e. radiographic assessments) is in line with current recommendations from Association of Urological Oncology (AUO) of the German Cancer Society (DKG) and display current routine in patients with advanced metastatic prostate cancer receiving systemic treatment. Therefore the risks of radiographic assessment may not be distinguished from the risks the patient would receive if not participating in the trial.
Assessments of hormonal changes of the pituitary-gonadal axis are based on blood samples taken at the time points for routine safety assessments under treatment with abiraterone acetate and therefore are considered no additional risk for the patients.
Overall risk/benefit assessment
The risks for patients participating in this trial are considered minimal with an acceptable risk/benefit ratio.

Statistical Analysis

Patient disposition and efficacy analyses will be performed on data from the intention-to-treat (ITT) population. All patients randomized into the study will be classified according to their assigned treatment group, regardless of the actual treatment received. The primary efficacy analyses will be on the ITT basis.

All patients who receive any part of abiraterone acetate will be included in the analysis of safety (Safety Population)

For this trial, the primary efficacy parameter will be the rate of radiographic progression free survival after 12 months. This is an exploratory phase-II trial where neither the primary endpoint radiographic progression-free survival (rPFS) nor the secondary endpoints will be powered for statistical significance. Each treatment arm will consist of 30 patients evaluable for the primary endpoint rPFS. Assuming a drop-out rate of 15% in each arm it is estimated that 70 patients need to be recruited for this trial at 12 sites across Germany. Accrual time for the trial is estimated to take 12 months.

Trial Timelines

Final protocol:	December	2013
Submission of protocol:	December	2013
First patient first visit:	August	2014
Last patient enrolled:	December	2015
Last patient last visit:	March	2017
Final report:	September	2017

Table of contents:

1 Background and introduction ... 13
1.1 Metastatic androgen independent prostate cancer ... 13
1.2 Abiraterone Acetate and the Active Metabolite Abiraterone ... 14
1.2.1 Dosing Rationale ... 15
1.2.2 Concurrent Prednisone ... 16
1.2.3 Status of Current Clinical Trials of Abiraterone Acetate ... 16
1.3 Rationale for Study Design and Control Group ... 17
1.4 Radiographic PFS as an Efficacy Endpoint ... 17
2 Objectives of the trial ... 18
2.1 Primary objective ... 18
2.2 Secondary Objectives ... 18
3 Risk/Benefit Analysis ... 19
3.1 Arm A (abiraterone acetate + prednisone + LHRH-therapy) ... 19
3.2 Arm B (abiraterone acetate + prednisone) ... 19
3.3 Risks for both treatment arms ... 19
3.4 Overall risk/benefit assessment ... 19
4 Investigational Plan ... 20
4.1 Overall Study Design and Plan ... 20
4.1.1 Primary Efficacy Endpoints ... 20
4.1.2 Secondary Endpoints ... 21
4.1.3 Safety Assessments ... 21
4.1.4 Study Duration and Dates ... 21
4.1.5 Study Timeplan ... 21
5 Study Population Selection ... 22
5.1 Study Population ... 22
5.2 Inclusion criteria ... 22
5.3 Exclusion Criteria ... 23
6 Study Treatment(s) ... 24
6.1 Description of Treatment(s) ... 24
6.1.1 Study Drug ... 24
6.1.2 Prednisone ... 24
6.1.3 LHRH-Therapy ... 24
6.2 Treatments Administered ... 24
6.2.1 Abiraterone Acetate ... 24
6.2.2 Prednisone ... 24

6.2.3 LHRH-therapy ... 24
6.3 Selection and Timing of Dose for Each Patient ... 24
6.4 Randomization Procedures ... 25
6.5 Concomitant Therapy ... 25
6.6 Prohibited Concomitant Medication Restrictions ... 26
6.7 Potential for Drug.-Drug Interactions ... 26
6.8 Drug accountability ... 26
6.9 Packaging and Labeling ... 26
6.10 Storage ... 27
6.10.1 Pharmacy Storage Requirements ... 27
6.10.2 Storage and Handling Requirements for the Patient ... 27
6.11 Investigational Product Retention and Accountability at Study Site ... 27
7 Study Procedures ... 27
7.1 Informed Consent ... 27
7.2 Medical History ... 28
7.3 Physical Examination ... 28
7.4 Clinical Laboratory Tests ... 28
7.4.1 Laboratory Parameters ... 28
7.4.2 Translational Research ... 29
7.5 Safety Data Collection, Recording, and Reporting ... 29
7.5.1 Definitions ... 29
7.5.2 Reporting Procedures for All Adverse Events ... 30
7.5.3 Severity of Adverse Events ... 31
7.5.4 Serious Adverse Event Reporting Procedures ... 31
7.5.5 Safety Reporting during Follow-Up ... 32
7.5.6 Pregnancy ... 32
7.5.7 Product Quality Complaint Handling ... 32
7.5.8 Abnormal Laboratory Results ... 32
7.5.9 Management of Study Drug-Related Events ... 33
7.6 Criteria for Discontinuation of Study Treatment ... 34
7.7 Withdrawal from Study ... 34
8 Study Activities ... 35
8.1 Baseline (Day -28 to Day 0) ... 35
8.2 Randomization ... 36
8.3 Treatment Period ... 36
8.3.1 Cycle 1 Day 1 ... 37

8.3.2 Cycle 1 Day 15 Visit 37
8.3.3 Cycle 2 and 3 Day 1 38
8.3.4 Cycle 4 Day 1 and any other following cycles 39
8.3.5 Cycles 4, 7, and 10 Day 1 (continue every 3rd cycle beyond Cycle 10, e.g. Cycles 13, 16, 19, 22...) 39
8.3.6 Treatment Discontinuation Visit 40
8.3.7 End-of-Treatment Visit 40
8.3.8 Post-Treatment Follow-up Period 40
9 Quality Control and Assurance 41
10 Planned Statistical Methods 41
10.1 General considerations 41
10.2 Determination of Sample Size 41
10.3 Analysis Populations 42
10.4 Interim Analysis 42
10.5 Demographics and Baseline Characteristics 42
10.6 Study endpoint(s) 43
10.6.1 Efficacy Endpoint(s) 43
10.7 Efficacy Analysis Population and Methods 43
10.8 Safety Evaluations 43
11 Steering committee 44
12 Administrative responsibilities 44
12.1 Investigators and Study Administrative Structure 44
12.1.1 Investigator Responsibilities 44
12.1.2 Protocol Adherence and Investigator Agreement 44
12.2 Independent Ethics Committee (EC) Approval 45
12.3 Ethical Conduct of the Study 45
12.4 Patient Information and Consent 45
12.5 Patient Confidentiality 45
12.6 Study Monitoring 46
12.7 Case Report Forms 46
12.8 Laboratory Assessments 46
12.9 Radiologic Review 46
12.10 Protocol Violations/Deviations 47
12.11 Access to Source Documentation 47
12.12 Retention of Data 47
12.13 Financial Disclosure 47
12.14 Study Publication Guidelines and Disclosures Policy 47

References 49
13 Appendices 51

Table of appendices:

Appendix 1: Schedule of Events
Appendix 2: Study Treatment Preparation and Dispension instructions
Appendix 3: Protocol-Specific PCWG2 Criteria
Appendix 4: Modified Response Evaluation Criteria in Solid Tumours (RECIST)
Appendix 5: National Cancer Institute Common Terminology Criteria for AEs
Appendix 6: ECOG Performance Status
Appendix 7: Creatinine Clearance
Appendix 8: Worst Pain within last 24 hours
Appendix 9: Protocol Definition of Chronic Administration of Opiate Analgesia
Appendix 10: Sponsor Signature
Appendix 11: Site Investigator Signature Page

Abbreviations

AA	Abiraterone Acetate
ACTH	Adrenocorticotropic hormone
ADL	Activities of Daily Living
AE	Adverse Event
ALK-P	Alkaline phosphatase
ALT	Alanin-amino-transferase
AST	Aspartat-amino-transferase
BP	Blood Pressure
BUN	Blood Urea Nitrogen
CALGB	Cancer and Leukemia Group B
CBC	Complete Blood Count
CI	Confidence Interval
CR	Complete Response
CRF	Case Report Form
CRPC	Castration-resistant prostate cancer
CT	Computed Tomography
CTC	Common Toxicity Criteria
CTCAE	Common Toxicity Criteria for Adverse Events
CYB5B	Cytochrome b5 type B
CYP11A1	Cytochrome P450 11A1
CYP17	Cytochrom P450 17
DHEA-S	Dehydroepandrosterone Sulphate
DHT	Dihydrotestosterone
DRE	Digital Rectal Examination
EC	Ethics Committee
ECG	Electrocardiogram
ECHO	Echodardiogram
ECOG	Eastern Cooperative Oncology Group
EDC	Electronic Data Capture
EP	European Pharmacopoeia
FSH	Follicle Stimulating Hormone
GCP	Good Clinical Practice
HIPAA	Health Information Portability and Accountability Act
HTN	Hypertension
HR	Hazard Ratio
3ßHSD	3ß-hydroxysteroid dehydrogenase
ICH	International Conference on Harmonisation
IDMC	Independent Data Monitoring Committee
IEC	Independent Ethics Committee
ITT	Intention-to-treat
IVF	Intravenous Fluids
LD	Longest Diameter
LDH	Lactate Dehydrogenase
LH	Luteinizing hormone
LHRH	Luteinizing hormone releasing hormone
MRI	Magnetic Resonance Imaging
NCI	National Cancer Institute
NYHA	New York Heart Association
OS	Overall Survival
PCWG2	Prostate-Cancer Working Group 2
PD	Progressive Disease

PFS	Progression-free Survival
PK	Pharmacokinetik
PQC	Product Quality Complaint
PR	Partial Response
PSA	Prostate Specific Antigen
PT	Preferred Term
RBC	Red Blood Cell (count)
RECIST	Response Evaluation Criteria in Solid Tumours
rPFS	Radiographic Progression-free Survival
SAE	Serious Adverse Event
SAER	Serious Adverse Event Report
SD	Stable Disease
SOC	System Organ Class
SRE	Skeletal Related Event
StAR	Steroidogenic Acute Regulatory Protein
SUSAR	Suspected Unexpected Serious Adverse Reaction
TPN	Total Parental Nutrition
ULN	Upper Limit of Normal
USP/NF	US Pharmacopeia-National Formulary
WBC	White Blood Cell (count)
WHO	World Health Organization

1 Background and introduction

1.1 Metastatic androgen independent prostate cancer

Treatment of patients with progressive castration-resistant prostate cancer (CRPC) with abiraterone in combination with prednisone lead to an increase in median survival of 4.6 months compared to prednisone alone in a large phase-III trial [1]. Furthermore progression-free survival of chemotherapy naïve patients was significantly improved by treatment with abiraterone and a strong trend toward improved overall survival was seen [2]. Inclusion of patients into the two phase-III and former trials required continuation of medical castration with LHRH-therapy in patients without prior surgical castration. Therefore the label of abiraterone (Zytiga®), at least in Germany and Switzerland contains an advice to continue LHRH-therapy during treatment with abiraterone. In castrate men treated with abiraterone while continuing on LHRH-therapy testosterone further decreased rapidly to undetectable levels [3]. This potential of abiraterone to achieve even a more complete androgen deprivation than LHRH-therapy appeals to speculate whether abiraterone may achieve and maintain testosterone deprivation at undetectable levels without concomitant LHRH therapy and that LHRH-therapy may be discontinued at initiation of therapy with abiraterone.

In general, cessation of LHRH-therapy in patients with castration-resistant prostate cancer is studied insufficiently and therefore current guidelines advice to continue medical castration [4]. These recommendations are mainly based on retrospective data and have never been investigated in prospective clinical trials. Nevertheless in current daily practice some patients discontinue LHRH-analogues, which even impaired recruitment to the abiraterone phase-III trials and the named patient program.

Although abiraterone may maintain androgen deprivation in the absence of LHRH-therapy, treatment without concomitant LHRH-therapy may impair efficacy of abiraterone due to effects of LHRH-therapy that are independent of androgen deprivation. Cessation of LHRH-therapy leads to rapid recovery of luteinizing hormone (LH) levels after a median of 58 days to 4.5 months, even after long-term medical castration [5,6]. This rise in LH levels is further enhanced since treatment with abiraterone in non-castrate mice induces a sustained increase in LH-levels despite suppression of testosterone [7]. This feedback mechanism was confirmed in non-castrate patients since treatment with abiraterone lead to an increase in LH-levels that returned to normal after cessation of therapy [8]. Apart from its action on the testes and adrenal glands, LH may, at least in part, act directly on prostate cancer cells via LH-specific receptors causing an increase in the expression of several key steroidogenic enzymes including StAR, CYB5B, CYP11A and 3ßHSD [9]. An induction of these enzymes has recently been shown in xenograft models with primary or secondary resistance against abiraterone and was deemed one possible cause of resistance [10]. The activation of these enzymes may enhance rescue pathways of testosterone and dihydrotestosterone production in prostate cancer cells [11] and thereby impair the efficacy of abiraterone.

Taken together, discontinuation of LHRH-therapy during treatment with abiraterone may impair efficacy of abiraterone and display an unpredictable hazard to the patients leading to early progression and consequently shorten cancer-specific and overall survival.

Investigation of the effects of discontinuing LHRH-therapy at the time of initiation of abiraterone treatment therefore displays a major medical need. This will be the first study to assess the efficacy of abiraterone acetate with and without concomitant LHRH-therapy in patients with CRPC. The results of this trial will gain evidence of the understanding of castrate- and hormone-resistant prostate cancer. Furthermore this trial may add evidence to the current recommendation to continue LHRH-therapy thereby improving the benefit of abiraterone treatment for the patient.

1.2 Abiraterone Acetate and the Active Metabolite Abiraterone

Abiraterone is [17-(3-pyridyl)androsta-5,16-dien-3ß-ol] andis a steroidal irreversible inhibitor of CYP17 (17α hydroxylase/C17,20 lyase), blocking 2 important enzymatic activities in the synthesis of testosterone (Figure1), based on the observation that nonsteroidal 3 pyridyl esters improved selectivity for inhibition of 17α-hydroxylase/C17,20 lyase[12]. Abiraterone is a potent inhibitor with an apparent inhibition constant of 0.5nM. Pharmacodynamic studies demonstrated that its effects on adrenal steroid synthesis were consistent with its mechanism of action. Antitumour effects were evident with PSA response and durable objective responses using Response evaluation criteria in solid tumours (RECIST) criteria in Phase I, II and III studies conducted to date [2,3,13,14].

Abiraterone acetate (CB7630) is the 3-acetate analog of abiraterone und thus a pro-drug of abiraterone. The chemical nomenclature of abiraterone acetate is 3ß acetoxy-17-(3-pyridyl)androsta-5,16-diene; its empirical formula is $C_{26}H_{33}NO_2$ and molecular weight is 391.55. Once absorbed after oral administration, abiraterone acetate is rapidly converted to the active form, abiraterone (Figure 2). Abiraterone was the predominant, if not the only metabolite of abiraterone acetate detected in blood both in preclinical studies [7] and in previously conducted clinical studies [8].

Figure1. The Enzyme Complexes Inhibited by Abiraterone [15]

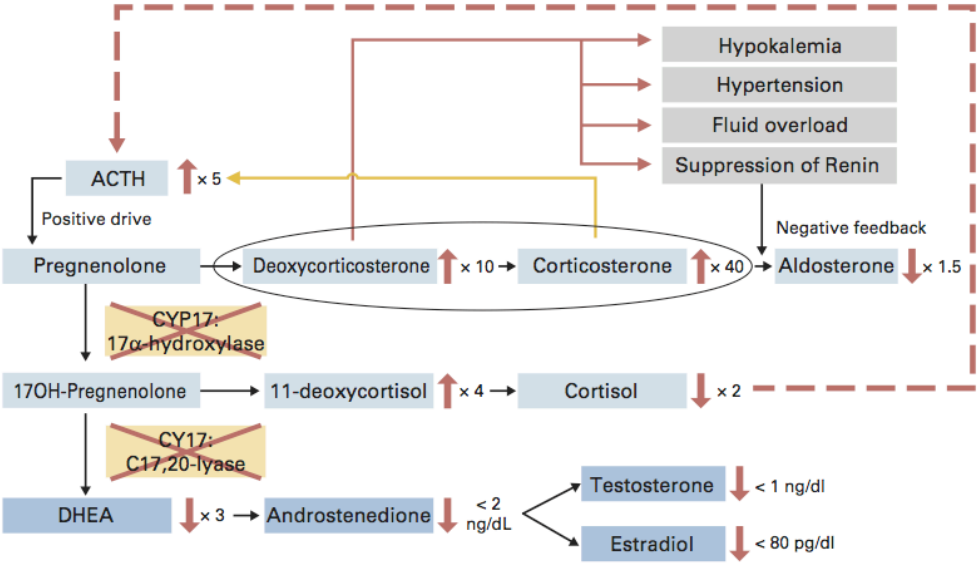

Figure 2 Prodrug Abiraterone Acetate is converted to Abiraterone after Absorption

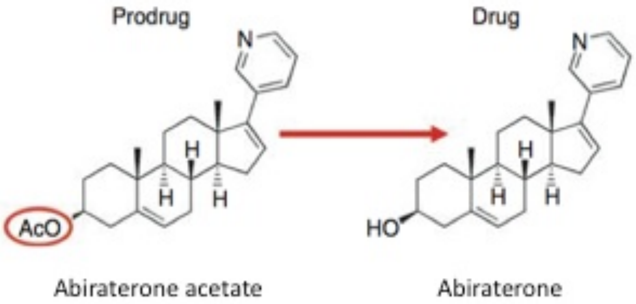

1.2.1 Dosing Rationale

The dose of abiraterone acetate (AA) in this study is 1000mg daily based on results of two Phase I dose-finding studies. In the first Phase I study with capsule formulation (COU-AA-001) [15], abiraterone acetate was evaluated for safety, pharmacokinetics, and its effects on adrenal steroid synthesis at dose levels ranging from 250mg to 2000mg. Preliminary analysis showed that abiraterone acetate was well tolerated at all dose levels. There were no hospital admissions related to study drug or any evidence of clinically significant adrenal insufficiency. Patients have been on this study for up to 18 months. In the second Phase I study (COU-AA-002) [3] that evaluated the safety and tolerability of abiraterone acetate tablet formulation at doses ranging from 250 to 1000mg, a daily dose of 1000mg has also been found to be safe and well tolerated.

Consistent with abiraterone acetate's mechanism of action, hypertension (HTN), hypokalemia, and lower extremity edema were the most commonly observed drug-related adverse events, which were all manageable with medication. Pharmacokinetik (PK) studies showed increased systemic drug exposure at higher doses. Adrenal metabolite analysis showed inhibition of CYP17 even at low doses of abiraterone acetate and a compensatory increase of corticosterone and deoxycorticosterone. Of note, antitumour activities were apparent at all dose levels tested. Data from dose-finding studies indicated that when PK, adrenal CYP17 inhibition, and efficacy signals are taken into consideration, the 1000mg dose offered consistent pharmacological effects without additional side effects. In the phase III trial (COU-AA-302) of chemotherapy-naïve patients, a favourable toxicity profile was reported (Table 1). Therefore, the 1000mg dose has been chosen for further efficacy and safety evaluation in this phase 2 study.

Adverse Event	Abiraterone–Prednisone (N=542)	Prednisone Alone (N=540)
	no. of patients (%)	
Any adverse event	537 (99)	524 (97)
Grade 3 or 4 adverse event	258 (48)	225 (42)
Any serious adverse event	178 (33)	142 (26)
Adverse event leading to treatment discontinuation	55 (10)	49 (9)
Adverse event leading to death*	20 (4)	12 (2)
Adverse event of grade 1–4 in ≥15% of patients in either group		
Fatigue	212 (39)	185 (34)
Back pain	173 (32)	173 (32)
Arthralgia	154 (28)	129 (24)
Nausea	120 (22)	118 (22)
Constipation	125 (23)	103 (19)
Hot flush	121 (22)	98 (18)
Diarrhea	117 (22)	96 (18)
Bone pain	106 (20)	103 (19)
Muscle spasm	75 (14)	110 (20)
Pain in extremity	90 (17)	85 (16)
Cough	94 (17)	73 (14)

Table 1. Adverse Events reported from the COU-AA-302 trial in patients with chemotherapy-naïve CRPC[2]. *The most common adverse events leading to death were general disorders, including disease progression, a decline in physical health, and infections including pneumonia and respiratory tract infection.

1.2.2 Concurrent Prednisone

In previous and ongoing clinical trials, patients receiving abiraterone acetate have been treated concurrently with glucocorticoids, including prednisone. Based on our understanding of the mechanism of abiraterone action and observations in patients with congenital deficiency of CYP17, we anticipated that a state of mineralocorticoid excess could occur after pharmacologic inhibition of CYP17. Resulting reduced corticoid levels may lead to a compensatory ACTH surge thereby resulting in hypertension, hypokalemia, and fluid retention (Figure1). Notably, patients with the rare syndrome of congenital CYP17 deficiency do not develop adrenocorticoid insufficiency, as corticosterone synthesis is unaffected. However, as corticosterone is a weaker glucocorticoid than cortisol, interruption of negative feedback control of adrenocorticotrophic hormone (ACTH) occurs, resulting in high levels of ACTH and steroid precursors upstream of CYP17 [16].

As expected, when abiraterone acetate was used as a single agent in Phase I and II studies, hypertension, hypokalemia, and fluid retention were observed and were primarily CTCAE grade 1-2 in severity. These side effects were readily managed with potassium supplementation, eplerenone (selective mineralocorticoid antagonist), antihypertensive agents, and low dose corticosteroids. Grade 1-2 fatigue was observed in some patients and was associated with discontinuation of corticosteroids as required per Phase II protocol entry criteria and extended duration of treatment with abiraterone acetate. Although there was no evidence of a dose-response relationship, administration of low dose corticosteroids as specified in the study improved symptoms of fatigue and tolerability of abiraterone acetate, including symptoms of mineralocortioid excess. The improved tolerability of abiraterone acetate after concomitant administration of low-dose corticosteroids was associated with suppression of ACTH surge and upstream adrenal steroids, suggesting that this combination may be a better-tolerated and safer regimen in this older and frail patient population. Prednisone was selected over other corticosteroids because it is commonly used as standard of care in combination with approved chemotherapy agents or as a monotherapy for palliation of symptoms. The safety and efficacy evaluation of abiraterone acetate with concurrently administered prednisone was continued in Phase II and III trials. The regimen of abiraterone acetate 1000mg daily and low dose prednisone 5mg bid has been chosen as the experimental intervention in this study.

1.2.3 Status of Current Clinical Trials of Abiraterone Acetate

Overall, as of 27 April 2014, over 800 subjects have been exposed to abiraterone acetate in Phase 2 studies and over 2,500 subjects in Phase 3 studies. Additionally, more than 7,500 subjects under the Named Patient Program and over 2,000 subjects under the Early Access Program received abiraterone acetate.

Results from Study COU-AA-301 were the basis for the marketing authorization of abiraterone acetate plus prednisone or prednisolone (hereafter referred to as prednisone) for mCRPC in subjects who had disease progression on or after docetaxel-based chemotherapy.

Results from Study COU-AA-302 were the basis of marketing authorization of abiraterone acetate plus prednisone for mCRPC in subjects who were asymptomatic or mildly symptomatic after failure of androgen deprivation therapy (ADT).

1.2.3.1 Chemotherapy-Naïve Castration-Resistant Prostate Cancer (CRPC)

Following safe expansion at the 1000 mg dose, the COU-AA-001 study enrolled additional patients to the Phase-2 portion of the study to further evaluate antitumour activity in chemotherapy-naïve CRPC patients. Chemotherapy-naïve men (n=54) with CRPC resistant to multiple hormonal therapies were treated in this two-stage phase I/II study. Declines in PSA ≥30%, ≥50% and ≥90% were observed in 43 (80%), 38 (70%) and 14 (24%) of patients respectively. Radiological evaluation reported disease regression by RECIST (CR or PR) in 52% of patients. Falls in circulating tumour cell counts [17], normalization of lactated dehydrogenase (LDH) and improving

symptoms with a reduction in analgesic use were commonly documented. Twenty-three patients have received abiraterone acetate capsules for more than 12 months as part of an extension protocol [18,19]. Similar response rates have been seen in COU-AA-002, a parallel phase I study investigating tablet formulations as opposed to capsules [3]. Most recently, the results of a large phase-III trial in patients with asymptomatic or mildly symptomatic CRPC patients without prior chemotherapy have been reported (COU-AA-302,[2]). In this trial, the median radiographic progression-free survival was 16.5 months with abiraterone plus prednisone and 8.3 months with prednisone alone (HR for abiraterone plus prednisone vs. prednisone alone = 0.53; 95% confidence interval [CI], 0.45 to 0.62; p<0.001). Over a median follow-up period of 22.2 months, overall survival was improved with abiraterone–prednisone (median not reached, vs. 27.2 months for prednisone alone; HR = 0.75; 95% CI, 0.61 to 0.93; p=0.01) but did not cross the efficacy boundary. Abiraterone plus prednisone showed superiority over prednisone alone with respect to time to initiation of cytotoxic chemotherapy, opiate use for cancer-related pain, prostate-specific antigen progression, and decline in performance status.

1.2.3.2 Castration-Resistant Prostate Cancer (CRPC) post Docetaxel

Apart from two earlier Phase 2 studies (COU-AA-003 and COU-AA-004) in post-docetaxel CRPC patients, abiraterone acetate has been evaluated in a large Phase 3 registration trial (COU-AA-301). The trial recruited 1195 patients who were randomly assigned (2:1 ratio) to receive either 1000mg abiraterone plus prednisone (797 patients) or placebo plus prednisone (398 patients)[14]. Median overall survival was 15.8 months in the abiraterone group and 11.2 months in the placebo group (HR = 0.74; p<0.0001) [20]. All the secondary end points analyzed provided support for the superiority of abiraterone acetate over placebo, including the confirmed PSA response rate (29% vs. 6%, P<0.001), the objective response rate on the basis of RECIST among patients with measurable disease at baseline (14% vs. 3%, P<0.001), time to PSA progression (10.2 months vs. 6.6 months), and median progression-free survival on the basis of radiographic evidence (5.6 vs. 3.6 months). On the basis of the PSA concentration, abiraterone acetate was associated with a 42% reduction in the risk of disease progression (HR = 0.58; 95% CI, 0.46 to 0.73; p<0.001), and on the basis of radiographic imaging, it was associated with a 33% reduction in the risk of progression (HR= 0.67; 95% CI, 0.58 to 0.78; p<0.001) [14].

1.3 Rationale for Study Design and Control Group

Given the risk that discontinuation of LHRH-therapy during treatment with abiraterone may impair efficacy of abiraterone and display an unpredictable hazard to the patients leading to early progression and consequently shorten cancer-specific and overall survival, the trial was designed as an exploratory two-arm, randomized, open-label phase II trial comparing the combination of AA + prednisone + LHRH-therapy vs. AA + prednisone alone. Blinding of the LHRH-therapy is not planned since it would mean to switch the patients after a long-term treatment with a specific LHRH-therapy to which the patient was adopted to a trial-specific LHRH-therapy that may cause side effects of which the causality or relationship may not be determined (i.e. being caused by either AA or the new LHRH-therapy). Therefore it is intended that patients randomized to the AA + prednisone + LHRH-therapy arm continue on the LHRH-therapy which they received prior to entering the trial.

1.4 Radiographic PFS as an Efficacy Endpoint

Clinical research in prostate cancer has been hindered by the difficulty in objectively defining cancer progression. Previous studies have used composite endpoints that included a mixture of patient reported, radiographic, and skeletal-related events. These composite endpoints attempted to address the complexity of clinical prostate cancer but suffered from subjectivity, difficulties in prospective definition, and were not amenable to independent review. Radiographic PFS as defined in this protocol is not a composite endpoint; each measurement is objective and associated with

clinical significance. The issues from using RECIST criteria alone to define radiographic progression in prostate cancer are well documented. Fewer than 50% of men with CRPC have measurable lesions greater than 2 cm in size, and most of these lesions are in lymph nodes which impact survival less than visceral metastases [21]. Conventional RECIST also lacks provisions to differentiate true progression of bone metastasis from the flare phenomen on bone scan due to healing of previous bone metastasis [22,23]. Although radiographic PFS has not been established as a strong predictor for overall survival in a small study of patients receiving docetaxel therapy, it was found to outperform PSA based progression [23]. Furthermore PFS (bone progression defined as two or more new lesions on bone scan, PSA progression using the PSA Working Group consensus criteria of 1999 [24], for objective progression in lung, liver, nodes, or soft tissue disease) at 3 and 6 months predicted overall survival (OS) in an analysis of different CALGB trials [25]. The COU-AA-301 trial also reported a benefit in radiographic progression free survival for abiraterone over placebo (5.6 vs. 3.6 months) [14] that showed a linear correlation with OS [26]. Furthermore, in this trial rPFS preceded PSA-progression in both treatment arms and is therefore of higher clinical value than PSA-progression to decide for treatment cessation.
Nevertheless, previous development programs (atrasentan [27] and docetaxel-treated patients [28]) have shown that two factors can limit the correlation of radiographic PFS with survival in CRPC.

- First, analyses of PFS have highlighted the difficulty in determining treatment effects based on PFS when the interval between assessments (12 weeks) was similar to the length of PFS. In contrast, results of the COU-AA-302 trial were able to report a significant difference in radiographic PFS between treatment groups using an interval of 12 weeks between assessments [14] that was predictive of OS. Therefore in this trial an interval between assessments of 12 weeks was chosen.
- Second, because early progression events could be confounded by tumour flare phenomenon, in this study new consensus guidelines will be applied to define progression requiring confirmatory bone scans at least six weeks after the original scan [29,30]. This will reduce the number of false positive determinations of CRPC progression.

Progression of metastatic bone disease is of paramount importance to CRPC patients since it is responsible for the severe morbidity of the skeletal related events such as fracture, pain, spinal cord compression, pre-emptive surgery or radiation for impeding fracture or spinal cord compression. In addition, painful bone metastasis often requires radiation, chemotherapy or radionuclide therapy [28]. Similarly, progression of soft tissue metastasis may be associated with clinically significant morbidity. Prevention of disease progression in pelvic and para-aortic lymph nodes reduces the risk for urinary outlet tract obstruction requiring invasive stent placement that is frequently complicated by infection. Development of liver metastasis could lead to organ failure and death. Thus, delay in cancer progression should result in clear clinical benefit to the patient.

In conclusion, rPFS as defined above is an objective and verifiable measurement likely to predict clinical benefit in the proposed study populations.

2 Objectives of the trial

2.1 Primary objective

The primary objective of the study is to analyze the clinical benefit of abiraterone acetate plus prednisone while sparing LHRH-therapy in chemotherapy-naïve patients with metastatic castration-resistant prostate cancer (CRPC).

2.2 Secondary Objectives

The secondary objectives of this study are:

- To establish additional clinically relevant information regarding early PSA responses to abiraterone and to correlate these with radiographic-progression free survival

- To investigate the value of the bone-scan index in the early course of treatment as a biomarker for response to treatment
- To investigate effects of both treatment arms on hormones of the pituitary-gonadal axis
- To characterize the safety profile of abiraterone acetate while sparing LHRH-therapy in comparison to continuing LHRH-therapy

3 Risk/Benefit Analysis

In accordance with the Declaration of Helsinki and the WHO recommendations for Good Clinical Practice (GCP) the trial is assessed for risks and benefits of patients who participate in the trial.

3.1 Arm A (abiraterone acetate + prednisone + LHRH-therapy)

Patients randomized to Arm A will receive the approved standard treatment for this indication. Therefore the risks of the therapy may not be distinguished from the risks of therapy the patient would receive if not participating in the trial.

3.2 Arm B (abiraterone acetate + prednisone)

Patients randomized to Arm B will receive less treatment compared to the approved standard treatment for this indication. The risks for the patients are only based on the effect of sparing LHRH-therapy on the primary endpoint of the trial, i.e. radiographic progression free survival. If the hypothesis of the trial holds true, patients in Arm B may experience radiographic progression earlier compared to patients randomized to Arm A. Shortening of time to radiographic progressioncannot not be predicted from the available results of studies conducted with abiraterone acetate. However, patients who experience radiographic progression are able to receive further treatment for advanced prostate cancer since several therapies have been approved for this indication (i.e. docetaxel, cabazitaxel, alpharadin). Therefore the risk on overall survival for patients participating in this trial is considered to be minimal.

In contrast, patients randomized to Arm B may benefit from sparing LHRH-therapy who therefore donot experience side effects from treatment related to the application of LHRH-therapy or toxicities.

3.3 Risks for both treatment arms

Risks that have to be taken into account for both treatment arms are radiographic assessments for the primary efficacy endpoint and drawing of blood samples for hormone analysis.

The Frequency of assessments of the primary study endpoint (i.e. radiographic assessments) is in line with current recommendations from Association of Urological Oncology (AUO) of the German Cancer Society (DKG) [31] and display current routine in patients with metastatic prostate cancer receiving systemic treatment. Therefore the risks of radiographic assessment may not be distinguished from the risks the patient would receive if not participating in the trial.

Assessments of hormonal changes of the pituitary-gonadal axis are based on blood samples taken at the time points for routine safety assessments under treatment with abiraterone acetate and therefore are considered no additional risk for the patients.

3.4 Overall risk/benefit assessment

The risks for patients participating in this trial are considered minimal with an acceptable risk/benefit ratio.

4 Investigational Plan

4.1 Overall Study Design and Plan

This is a national exploratory Phase 2 multicenter, randomized, open-label study with a randomization allocation ratio of 1:1 [abiraterone acetate + prednisone + LHRH-therapy(Arm A) versus abiraterone acetate + prednisone (Arm B)]. This study will be conducted at approximately 12 investigative sites in Germany (academic hospitals and office based urologists/oncologists) and 70 patients will be enrolled. A complete list of participating centres can by found at www.spare-trial.de.

4.1.1 Primary Efficacy Endpoints

Primary endpoint of the study is rate of radiographic progression-free survival (rPFS) at month 12 based on parameters suggested by PCWG2 [29] and modified RECIST as the time from randomization to the occurrence of one of the following:

1. A patient is considered to have progressed by bone scan if:
   a. The first bone scan with ≥2 new lesions compared to baseline is observed <12 weeks from randomization and is confirmed by a second bone scan taken ≥6 weeks later shown ≥2 additional new lesions (a total of ≥4 lesions compared to baseline);
   b. The first bone scan with ≥2 new lesions compared to baseline is observed ≥12 weeks from randomization and the new lesions are verified on the next bone scan ≥6 weeks later (a total of ≥2 new lesions compared to baseline).
2. Progression of soft tissue lesions measured by CT or MRI as defined in modified RECIST criteria.
3. Death from any cause

In the analysis of rPFS, the following censoring rules apply:

1. If the patient does not a have baseline scan or on-study scans, the patient will be censored on the date of randomization
2. If the patient does not show progression according to modified RECIST, the patient will be censored on the date of the last scheduled scan
3. Bone scan censoring rules
   a. if the patient remains on study treatment and prior scans do not show radiographic progression, the patient will be censored on the date of the last scan showing no disease progression
   b. if the patient discontinues study treatment for any reason and progression was not observed in the scans prior to discontinuation, the patient will be censored on the last scan showing no disease progression
   c. if the patient discontinues study treatment for any reason and additional new lesions were observed in the scan prior to the discontinuation, and there was no confirmatory scan, the patient will be censored on the date of the last scan that showed no disease progression
4. Patients will also be censored on the date of the last scan that shows no disease progression if:
   a. the patient receives another therapy known or intended for treatment of metastatic CRPC during the study
   b. the patient misses ≥2 planned radiographic scans or has ≥2 consecutive unreadable scans

c. the patient has unequivocal progression of non-bone non-target lesions (eg, appearance of non-measurable visceral metastases or pathologically confirmed malignant effusions).

4.1.2 Secondary Endpoints

Secondary endpoints of the study include

- PSA response rate scored in patients achieving a post-treatment PSA decline of at least 50% according to the protocol-specific PCWG2 criteria
- Time to PSA-progression will be measured from the time interval from the date of randomization to the date of the PSA progression as defined in the protocol-specific PCWG2 criteria. The determination of PSA progression will require that the patient receive at least 3 cycles of therapy.
- Objective response rate in patients with measurable disease (RECIST)
- Value of the bone-scan index as a biomarker of response to treatment
- Changes in pituitary gonadal axis by measurement of androgens and hormones (LHRH, LH, FSH, testosterone, DHT)
- Safety

4.1.3 Safety Assessments

- Medical history, vital sign measurements, physical examination, and body weight
- Concomitant therapy and procedures
- Adverse events (AEs) and serious adverse events (SAEs) including laboratory test. AEs will be graded and summarized according to the National Cancer Institute (NCI) Common Toxicity Criteria for Adverse Events (CTCAE), version 4.0 (Appendix 5)
- Blood chemistry and hematology
- Electrocardiograms (ECGs) and cardiac ECHO (only in patients with known or suspicion of heart failure (heart failure, uncontrolled hypertension, and history of myocardial infarction).

4.1.4 Study Duration and Dates

The study period will consist of screening, treatment, and follow-up phases (see section 8: Study Activities). In this study, patients will receive study treatment (abiraterone acetate plus LHRH-therapy or abiraterone acetate) plus prednisone until radiographic progression of disease and/or unequivocal clinical progression as defined in section7.6.

4.1.5 Study Timeplan

The following timelines are planned for the study:

- Protocol finalisation:	December 2013
- Site selection:	September, 2013
- Submissions to CA and ECs:	December 2013
- Positive Vote and Permission:	April 2014
- Site initiations:	April-June 2014
- First Patient First Visit:	August 2014
- Last Patient First Visit:	December 2015
- End of Treatment Phase:	March2017

- Last Patient Last Visit: March 2017
- Study Report: September 2017
- Publication of results 2017 ff

5 Study Population Selection

5.1 Study Population

Altogether, 70 medically castrated male chemotherapy-naïve patients with metastatic CRPC will be enrolled from 12 German study sites.

5.2 Inclusion criteria

Each patient must meet the following criteria to be enrolled in this study:

1. Willing and able to provide written informed consent
2. Written Data Protection Consent has been obtained
3. Male aged 18 years and above
4. Histologically or cytologically confirmed adenocarcinoma of the prostate
5. Metastatic disease documented by positive CT/MRI and/or bone scan (both must be performed). If lymph node metastasis is the only evidence of metastasis, it must be ≥2 cm in diameter
6. Prostate cancer progression documented by PSA according to PCWG2 or radiographic progression according to modified RECIST criteria
7. Asymptomatic or mildly symptomatic from prostate cancer. A score of 0-1 for the question of worst pain within last 24 hours (Appendix 8) will be considered asymptomatic, and a score of 2-3 will be considered mildly symptomatic.
8. Medically castrated, with testosterone levels of <20-50 ng/dl (< 2.0 nM).
9. Combined androgen blockade is permitted, but not required.If patients received combined androgen blockade with an anti-androgen they must have shown PSA progression after discontinuing the anti-androgen prior to enrolment (≥4 weeks since last flutamide, ≥6 weeks since last bicalutamide or nilutamide).
10. Eastern Cooperative Oncology Group (ECOG) Performance Status of ≤2 (Appendix 6)
11. Hemoglobin ≥9.0 g/dL independent of transfusion
12. Platelet count ≥100,000/µl
13. Serum albumin ≥3.0 g/dl
14. Serum creatinine < 1.5 x ULN or a calculated creatinine clearance ≥60 ml/min (Appendix 7)
15. Serum potassium ≥3.5 mmol/l
16. Liver function:

a. Serum bilirubin <1.5 x ULN (except for patients with documented Gilbert's disease)

b. AST or ALT <2.5xULN

17. Able to swallow the study drug whole as a tablet
18. Life expectancy of at least 6 months
19. Patients who have partners of childbearing potential must be willing to use a method of birth control with adequate barrier protection as determined to be acceptable by the principal investigator and sponsor during the study and for 13 weeks after last study drug administration.

5.3 Exclusion Criteria

1. Surgical castration (i.e. orchiectomy).
2. Application of any LHRH-therapy (LHRH-analogue or LHRH-antagonist) within 3months (for patients receiving a 3-months formulation) or 1 months (for patients receiving a 1-month formulation) prior to Cycle 1 day 1.
3. Patients receiving a 6- or 12-months formulation of LHRH-therapy
4. Active infection or other medical condition that would make prednisone/prednisolone (corticosteroid) use contraindicated
5. Any chronic medical condition requiring a higher dose of corticosteroid than 5mg prednisone/prednisolone bid.
6. Pathological finding consistent with small cell carcinoma of the prostate
7. Liver or visceral organ metastasis
8. Known brain metastasis
9. Use of opiate analgesics for cancer-related pain, including codeine, tramadol, tilidin and others (see Appendix 9), currently or anytime within 4 weeks of Cycle 1 Day 1.
10. Prior cytotoxic chemotherapy or biologic therapy for the treatment of CRPC
11. Radiation therapy for treatment of the primary tumour within 6 weeks of Cycle 1, Day 1
12. Radiation or radionuclide therapy for treatment of metastatic CRPC
13. Prior treatment with Abiraterone acetate or other CYP17 inhibitors (ketoconazole, TAK700, TOK001), Enzalutamide (Xtandi) or investigational agents targeting the androgen receptor for prostate cancer for more than 7 days
14. Prior systemic treatment with an azole drug (e.g. fluconazole, itraconazole) within 4 weeks of Cycle 1, Day 1
15. Prior flutamide (Eulexin) treatment within 4 weeks of Cycle 1, Day 1 (patients whose PSA did not decline for three or more months in response to antiandrogen given as a second line or later intervention will require only a two week washout prior to Cycle 1, Day 1)
16. Bicalutamide (Casodex), nilutamide (Nilandron) within 6 weeks of Cycle 1 Day 1 (patients whose PSA did not decline for three or more months in response to antiandrogen given as a second line or later intervention will require only a two week washout prior to Cycle 1, Day 1)
17. Uncontrolled hypertension (systolic BP ≥160 mmHg or diastolic BP ≥95 mmHg). Patients with a history of hypertension are allowed provided that blood pressure is controlled by anti-hypertensive treatment
18. Active or symptomatic viral hepatitis or chronic liver disease
19. History of pituitary or adrenal dysfunction
20. Clinically significant heart disease as evidenced by myocardial infarction, or arterial thrombotic events in the past 6 months, severe or unstable angina, or New York Heart Association (NYHA) Class II-IV heart disease or cardiac ejection fraction measurement of <50% at baseline
21. Any condition that requires treatment with Digoxin, digitoxin, and other digitalis drugs
22. Atrial Fibrillation, or other cardiac arrhythmia requiring therapy
23. Other malignancywith a ≥30% probability of recurrence within 24 months, except non-melanoma skin cancer.
24. Administration of an investigational therapy within 30 days of Cycle 1, Day 1
25. Any condition, which, in the opinion of the investigator, would preclude participation in this trial.

6 Study Treatment(s)

6.1 Description of Treatment(s)

6.1.1 Study Drug

Abiraterone acetate 250 mg tablets are oval, white to off-white and contain abiraterone acetate and compendial (USP/NF/EP) grade lactose monohydrate, microcrystalline cellulose, croscarmellose sodium, povidone, sodium lauryl sulfate, magnesium stearate, colloidal silicon dioxide, and purified water, in descending order of concentration (the water is removed during tabletting).Abiraterone acetate 250 mg tablets will be available in bottles of 120 tablets.

Abiraterone acetate is marketed but not approved for the treatment under study.Refer to the Investigator's Brochure for additional information. In this study, abiraterone acetate will be provided by Janssen-Cilag.

6.1.2 Prednisone

Prednisone (5 mg tablets bid) will be prescribed as marketedand will be applied orally

6.1.3 LHRH-Therapy

LHRH-therapy, either as LHRH-analogues or LHRH-antagonists, will be prescribed as marketed. Patients randomized to the AA + Pred + LHRH-therapy will continue on the same LHRH-therapy they received prior to entering the trial. Patients should stay on the same LHRH-therapy throughout the treatment phase.

6.2 Treatments Administered

6.2.1 Abiraterone Acetate

Patients will be instructed to take 4 tables (abiraterone acetate) orally (per os) at least 1 hour before a meal or 2 hours after a meal any time up to 10 pm every day.

6.2.2 Prednisone

Patients will be instructed to take 5 mg prednisone or prednisolone per os, twice daily.

6.2.3 LHRH-therapy

Patients who are randomized to the AA + Pred + LHRH-therapy arm (Arm A) will continue on the same LHRH-therapy that had been used prior to randomization. LHRH-therapy (LHRH-analogue or LHRH-antagonist) will be administered according to the labeling information of the specific drug.

6.3 Selection and Timing of Dose for Each Patient

All patients must commence treatment within 72 hours (3 calendar days) of randomization. Each treatment cycle consists of 28 consecutive days. Patients may take study treatment (AA + Pred + LHRH-therapy or AA + Pred) until radiographic progression and/or unequivocal clinical progression of disease (as defined in Section 7.6), at which time study treatment will be discontinued. If the patient had radiographic progression but no unequivocal clinical progression, and alternate treatment is not initiated, the patient may continue on study treatment at the Investigator's discretion (see Section 7.6). If study treatment will be discontinued, the dose of prednisone will be gradually reduced if clinically indicated.

It is not required for the prednisone to be taken with study treatment (abiraterone acetate) at the same time. The dose of prednisone will remain unchanged in the event that abiraterone dose is changed. If a prednisone dose is missed, it should be omitted and will not be made up.

6.4 Randomization Procedures

Patients will be randomized after the investigator has verified that all eligibility criteria have been met. Patients will be randomized to receive abiraterone acetate plus prednisone and LHRH-Therapy or prednisone alone in a 1:1 ratio. No patient may be randomized to receive a treatment number prior to confirmation of diagnosis of prostate carcinoma. This is an open-label study so that blinding of treatments needs not to be done.

Randomization will take place across all study sites by online randomization in the eCRF-database. At randomization, a unique patient identification number will be assigned to each patient. The patient's identification number will be used on all study-related documents including case report forms (eCRFs). Patient identification numbers will not be reused.

6.5 Concomitant Therapy

The use of any concurrent drug from screening and while on study, prescription or over-the-counter, is to be recorded on the patient's CRF along with the reason the drug was taken.

Concurrent enrollment in another clinical investigational drug or device study is prohibited.

Supportive care medications are permitted with their use following institutional guidelines.

The following supportive care medications are considered permissible during the study:

- Conventional multivitamins, selenium and soy supplements
- Additional systemic glucocorticoid administration such as "stress dose" glucocorticoid is permitted if clinically indicated for a life threatening medical condition, and in such cases, the use of steroids will be documented as concomitant drug
- Bisphosphonates and denosumab usage is allowed only if patients are on the medication prior to Study Day 1
- Transfusions and hematopoietic growth factors per institutional practice guidelines
- If the permissibility of a specific drug/treatment is in question, please contact the study sponsor

The following interventions are permissible if the patient has a documented progression event but has not met all three criteria (Section 7.6) for discontinuation of treatment:

- Palliative Radiation – one course of involved field radiation (single or multi-fraction) to a single site; radiation to more than one site of disease will NOT be permitted
- Bisphosphonates – addition of a bisphosphonate or changing the type of bisphosphonates will only be allowed if a new skeletal related event (SRE) or bone progression is documented
- Glucocorticoids – an increase in the dose of prednisone or prednisolone or addition of a more potent glucocorticoid such as dexamethasone to treat prostate cancer related signs and symptoms, such as fatigue and pain, will be considered a disease progression event.

6.6 Prohibited Concomitant Medication Restrictions

The concurrent administration of other anticancer therapy, including cytotoxic, hormonal (except of LHRH-therapy in the appropriate treatment group), or immunotherapy, is prohibited during study treatment Phase. Use of other investigational drug therapy for any reason is prohibited.

Concomitant therapy with any of the following listed is restricted:

- 5α-reductase inhibitor
- Chemotherapy
- Immunotherapy
- Ketoconazole, diethylstilbestrol, PC-SPES, MDV3100, TAK700, TOK001, and other preparations such as saw palmetto thought to have endocrine effects on prostate cancer
- Radiopharmaceuticals such as strontium (^{89}Sr), samarium (^{153}Sm) or alpharadin (radium-223 Chloride)
- Aldactone, Spironol (spironolactone)
- Digoxin, digitoxin, and other digitalis drugs
- Cyproterone acetate
- Fludrocortisone acetate (Florinef)

The decision to administer a prohibited drug/treatment should be made based on the consideration of the safety of study participant.

Patients who require the use of any of these agents will be discontinued from study-treatment phase and will enter the follow-up phase.

6.7 Potential for Drug.-Drug Interactions

Investigators should keep in mind the possibility that abiraterone acetate may interact with concomitant medications. For detailed and updated information please refer to the investigators brochure. If at any time an investigator suspects a drug-drug interaction due to abiraterone acetate therapy, an adverse event report should be filed with the sponsor (see 7.5.1.1). Additional information is provided in the abiraterone acetate labeling information.

6.8 Drug accountability

A current and accurate account of the number of investigational tablets the investigator received from B&C, dispensed to the patients, the number of units returned to the investigator by the patient, and the number of units returned to B&C or its representative or destroyed on site during and at the completion of the study must be maintained. A detailed inventory must be completed for the study treatment. Unused study treatments are only allowed to be destroyed after the monitor checked accountability and has given written permission.

6.9 Packaging and Labeling

Abiraterone acetate tablets will be provided to each site. Patients will be provided with a 30-day supply to allow for visits to occur every 28 days with a ± 2 days window.

Information presented on the labels for investigative product will comply with applicable local regulations.

Site pharmacist or designated site personnel will dispense the study treatment to each patient in accordance with this protocol under the guidelines of the site's dispensation standard operating procedure.

6.10 Storage

6.10.1 Pharmacy Storage Requirements

The study treatment must be stored in a secure area and administered only to patients entered into the clinical study in accordance with the conditions specified in this protocol.

Bottles of study treatment should be stored at a room temperature between 15°-30°C with the cap on tightly and should not be refrigerated. Additional information is provided in the labeling information.

6.10.2 Storage and Handling Requirements for the Patient

Bottles of study treatment should be stored at room temperature with the cap on tightly and should not be refrigerated. Patients should be advised to keep all medications out of the reach and out of sight of children. Furthermore Patients should be advised that women with childbearing potential and pregnant women should handle the abiraterone tablets only while wearing gloves.

6.11 Investigational Product Retention and Accountability at Study Site

At the time of delivery of study treatment to the site, the investigator, designee, or Pharmacist (where appropriate) will sign a drug receipt form to confirm that the supplies for the study have been received. This form will specify supply, lot numbers, quantities shipped/delivered, and date of receipt. The form will also contain statements confirming that the study treatment has been received in good condition.

Study treatment must be stored in a secure location at room temperature between 15°-30°C. Accountability for study treatment is the responsibility of the investigator. More details concerning this responsibility are included inAppendix 2.

Study treatment must only be dispensed by a Pharmacist or medically qualified staff. Study treatment is to be dispensed only to patients enrolled in this study. Once the study treatment is prepared for a patient, it can only be administered to that patient.

The study site must maintain accurate records demonstrating dates and amount of study treatment (abiraterone) received, to whom dispensed (patient by patient accounting), and accounts of any study treatment accidentally or deliberately destroyed. At the end of the study, reconciliation must be made between the amounts of study treatment supplied.

Study site staff should refer to Appendix 2 and the labeling information for specific instructions on the handling, storage, and administration of the study drug.

7 Study Procedures

7.1 Informed Consent

A signed, Ethics Committee (EC) approved, informed consent must be obtained from patients. Confirmation of the patient's informed consent and the informed consent process must also be documented in the patient's medical record.

A copy of the fully signed informed consent will be given to the patient.

7.2 Medical History

Medical history, such as previous treatments, procedures, and conditions will be collected during the screening period.

7.3 Physical Examination

Evaluations should be performed by the same evaluator throughout the study whenever possible. If it is not possible to use the same evaluator to follow the patient, then evaluations should overlap (i.e., examine the patient together and discuss findings) for at least one visit.

Physical examination includes HEENT (head, eyes, ears, nose, and throat), chest, cardiac, abdominal, extremities, neurologic, and lymph node examinations. Weight will be recorded at every visit. Height will be recorded at screening visit only. Vital signs include upright blood pressure, heart rate, and oral or aural body temperature.

7.4 Clinical Laboratory Tests

7.4.1 Laboratory Parameters

Clinical laboratory tests will be done in local labs at every study site and include the following:
Table 2: **List of Laboratory Tests**

Hematology:
- Hematocrit (Kct)
- Hemoglobin (Hgb)
- Platelet count
- Red blood cell (RBC) count with differential
- White blood cell (WBC) count with differential

Serum Lipids
- Cholesterol
- HDL
- LDL
- Triglycerides

Tumour markers
- Prostate specific antigen (PSA)

Additional laboratory tests:
- Serum Testosterone*
- DHEA-S*
- Luteinizing Hormone (LH)*
- Luteinizing Hormone Releasing Hormone (LHRH)*
- Follicle Stimulating Hormone (FSH)*
- Dihydrotestosterone (DHT)*

Serum Chemistry:
- Albumin (ALB)
- Alkaline phosphatase (ALK-P)
- Alanine aminotransferase (ALT; SGPT)
- Aspartate aminotransferase (AST; SGOT)
- Blood urea nitrogen (BUN)
- Calcium (Ca)
- Chloride (Cl)
- Creatinine
- Gamma-glutamyl transferase (GGT)
- Glucose
- Lactate dehydrogenase (LDH)
- Magnesium
- Potassium (K)
- Sodium (Na)
- Total bilirubin
- Total protein
- Uric acid

***These parameters will be shipped to and analyzed in a central lab (see 7.4.2)**

7.4.2 Translational Research

Blood sampled collected at the study sites will be used for study-specific additional laboratory tests (see Table 2: **List of Laboratory Tests**). Collection and storage of collected blood samples will be approved by subject consent and by the independent ethic committee. Samples will be stored at the Department of Urology, Saarland University Medical Center.

Collected blood samples will be destroyed at the completion of the study.

7.5 Safety Data Collection, Recording, and Reporting

All observed or volunteered adverse events regardless of treatment group or causal relationship to study drug will be recorded on the adverse event page(s) of the case report form (CRF).

7.5.1 Definitions

7.5.1.1 Adverse Events

An adverse event is defined in the International Conference on Harmonisation (ICH) Guideline for Good Clinical Practice as "any untoward medical occurrence in a patient or clinical investigation subject administered a pharmaceutical product and that does not necessarily have a causal relationship with this treatment." (ICH E6:1.2).

For all adverse events, the investigator must pursue and obtain information adequate to both determine the outcome of the adverse event and to assess whether it meets the criteria for classification as a serious adverse event (see Section 7.5.1.2). For all adverse events, sufficient information should be obtained by the investigator to determine the causality of the adverse event. The investigator is required to assess causality. For every adverse event, follow-up by the investigator is required until the event resolves or stabilizes at a level acceptable to the investigator and the sponsor or its designated representative.

Adverse event will be recorded until end-of-treatment visit has been performed. No further recording of adverse events is necessary.

Interventions for pretreatment conditions (e.g., elective cosmetic surgery) or medical procedures that were planned before study enrollment are not considered adverse events.

7.5.1.2 Serious Adverse Events

A serious adverse event (SAE) is defined as an adverse event that:

- Results in death;
- Is life threatening (places the subject at immediate risk of death);
- Requires in-patient hospitalization or prolongation of existing hospitalization;
- Results in persistent or significant disability/incapacity;
- Is a congenital anomaly/birth defect

Important medical events that may not result in death, be life threatening, or require hospitalization may be considered as SAE when, based upon appropriate medical judgment, they may jeopardize the patient and may require medical or surgical intervention to prevent one of the outcomes listed in this definition. Examples of such medical events include allergic bronchospasm requiring intensive treatment in an emergency room or at home, blood dyscrasias or convulsions that do not result in inpatient hospitalization, or the development of drug dependency or drug abuse.

A hospitalization meeting the definition for "serious" is any inpatient hospital admission that includes a minimum of an overnight stay in a health care facility.

Inpatient admission does not include: rehabilitation facilities, hospice facilities, skilled nursing facilities, nursing homes, routine emergency room admissions, same day surgeries (as outpatient/same day/ambulatory procedures), or social admission (e.g., subject has no place to sleep).

7.5.1.3 Suspected Unexpected Serious Adverse Reactions (SUSARS)

Suspected Unexpected Serious Adverse Reactions (SUSARS) are events which are serious as per the above criteria, the nature or severity of which is not consistent with the applicable product information (e.g., Investigator's Brochure) and are judged by the investigator or the sponsor to be at leastpossiblyrelated to study treatment.

7.5.2 Reporting Procedures for All Adverse Events

The investigator is responsible for ensuring that all adverse events (as defined in Section 7.5.1 and as further specified below) observed by the investigator or reported by subjects are collected and recorded in the subjects' medical records, in the CRF, and, for serious adverse events, on the serious adverse event report (SAER) form. These adverse events will include the following:

- All serious adverse events (as defined in Section 7.5.1.2) that occur after the subject has signed the informed consent form and up to 30 days after the last dose will be documented.

- All non-serious adverse events (as defined in Section 7.5.1) that occur after randomization to study treatment up to 30 days after the last dose will be documented.

The following adverse events attributes must be assigned by the investigator: adverse event diagnosis or syndrome(s) (if known, signs or symptoms if not known); event description (with detail appropriate to the event); dates of onset and resolution; severity; assessment of relation to study treatment; action taken; and outcome. The investigator may be asked to provide follow-up information, discharge summaries, and extracts from medical records or CRFs.

For all adverse events, sufficient information should be obtained by the investigator to determine the causality of the adverse event (e.g. study drug or other illness). The relationship of the adverse event to the study treatment will be assessed following the definitions below:

- **Not Related**: An adverse event that is not related to the use of the drug.

- **Doubtful:** An adverse event for which an alternative explanation is more likely, eg, concomitant drug(s), concomitant disease(s), or the relationship in time suggests that a causal relationship is unlikely.

- **Possible:** An adverse event that might be due to the use of the drug. An alternative explanation, eg, concomitant drug(s), concomitant disease(s), is inconclusive. The relationship in time is reasonable; therefore, the causal relationship cannot be excluded.

- **Probable:** An adverse event that might be due to the use of the drug. The relationship in time is suggestive (eg, confirmed by dechallenge). An alternative explanation is less likely, eg, concomitant drug(s), concomitant disease(s).

- **Very Likely:** An adverse event that is listed as a possible adverse reaction and cannot be reasonably explained by an alternative explanation, eg, concomitant drug(s), concomitant disease(s). The relationship in time is very suggestive (eg, it is confirmed by dechallenge and rechallenge).

7.5.3 Severity of Adverse Events

- Adverse event severity is a clinical determination of the intensity of an AE and SAEs. The severity assessment for an AE/SAE should be completed using the NCI Common Terminology Criteria for Adverse Events (CTCAE, version 4.0).

Any AE/SAE not listed in the CTCAE version 4.0 will be graded as follows:

SEVERITY OF EVENT	
Grade	Definition
1	Mild: Symptoms which do not interfere with patient's daily activities
2	Moderate: Symptoms which may interfere with patient's daily acitivities
3	Severe: Events which interrupt patient's usual daily acitivities
4	Life-threatening or disabling.
5	Death

Medically significant adverse events considered related or unrelated to the study treatment by the investigator or the sponsor will be followed until resolved or considered stable.

It will be left to the investigator's clinical judgment to determine whether an adverse event is related and of sufficient severity to require the subject's removal from treatment or from the study. A subject may also voluntarily withdraw from treatment due to what he perceives as an intolerable adverse event. If either of these situations arises, the subject should be strongly encouraged to undergo an End of Study Treatment assessment and be under medical supervision until symptoms cease or the condition becomes stable.

7.5.4 Serious Adverse Event Reporting Procedures

Serious adverse events will be collected and recorded at least throughout the study period, beginning with the signing of the informed consent through 30 days after the end of the treatment phase.

All serious adverse events that occur after the subject has signed the informed consent from or during the study must be reported within 24 hours of discovery or notification of the event to the sponsor, its designated representative or study personnel through the planned adverse event reporting process and recorded in the appropriate case report form (CRF).

Relevant medical records should be provided to the sponsor as soon as they become available; autopsy reports should be provided for deaths if available.

Related serious adverse events are events that are judged by the investigator or by the sponsor to be at leastpossibly related to study treatment. See definitions above.

Determination of expectedness will be based on the contents of the Investigator's Brochure.

If a subject is permanently withdrawn from the study because of a serious adverse event, this information must be included in the initial or follow-up Serious Adverse Event Report Form as well as the End of Study Treatment Case Report Form.

The sponsor is responsible for notifying the EC and competent authorities of SUSARs occurring at the sites. Relevant information regarding SUSARs occurring during the study will be forwared to all study sites and investigators according to the current recommendations of the national pharmaceutical authorities (BfArM). Annual safety reports will be prepared by the sponsor according to the current guidelines for good clinical practice (GCP).

7.5.5 Safety Reporting during Follow-Up

For the follow-up phase after end of study treatment, seriousand non-serious adverse events (SAEs and AEs)considered at least possibly related to investigational drug will be collected and reported on the Serious Adverse Event Report form to the sponsor within 24 hours of discovery or notification of the event.

7.5.6 Pregnancy

Because the study drug may have an effect on sperm, or if the effect is unknown, pregnancies in partners of male subjects included in the study will be reported by the investigational staff within 24 hours of their knowledge of the event. Abnormal pregnancy outcomes are considered serious adverse events and must be reported using the Serious Adverse Event Form.

Follow-up information regarding the outcome of the pregnancy and any postnatal sequelae in the infant will be required.

7.5.7 Product Quality Complaint Handling

A product quality complaint (PQC) is defined as any suspicion of a product defect related to manufacturing, labeling, or packaging, i.e. any dissatisfaction relative to the identity,quality, durability, reliability of a product, including its labeling or package integrity. PQCs may have an impact on the safety and efficacy of the product. Timely, accurate,and complete reporting and analysis of PQC information from clinical studies are crucial for the protection of subjects, investigators, and the sponsor, and are mandated byregulatory agencies worldwide.

7.5.8 Abnormal Laboratory Results

All clinically important abnormal laboratory tests occurring during the study will be repeated at appropriate intervals until they return either to baseline or to a level deemed acceptable by the investigator and the sponsor (or his/her designated representative), or until a diagnosis that explains them is made. The criteria for determining whether an abnormal laboratory test result should be reported as an adverse event are as follows:

1. Test result is associated with accompanying symptoms, and/or
2. Test result requires additional diagnostic testing or medical/surgical intervention (merely repeating an abnormal test, in the absence of any of the above conditions, dose not meet criteria for reporting an AE), and/or
3. Test result leads to a change in the study dosing or discontinuation from the study, significant additional concomitant drug treatment or other therapy, and/or
4. Test result leads to any of the outcomes included in the definition of a serious adverse event, and/or
5. Test result is considered to be an adverse event by the investigator or sponsor

Any abnormal test result that is determined to be an error does not require reporting as an adverse event, even if it did meet one of the above conditions except for condition #4. Clinically significant laboratory results must be recorded in the patient's CRF.

7.5.9 Management of Study Drug-Related Events

In clinical studies in subjects with mCRPC, the most common adverse events related to abiraterone acetate have included fatigue most likely attributable to the underlying disease; and hypertension, hypokalemia, fluid retention/edema, and due to mineralocorticoid excess caused by compensatory ACTH drive. In this study, low-dose prednisone is expected to mitigate these effects through abrogation of the ACTH drive.

Following prolonged therapy with corticosteroids, subjects may develop Cushings syndrome characterized by central adiposity, thin skin, easy bruising, and proximal myopathy. Withdrawal of the corticosteroid may result in symptoms that include fever, myalgia, fatigue, arthralgia, and malaise. This may occur even without evidence of adrenal insufficiency.

In the event where dose-reduction is used for management of adverse events other than hepatotoxicity, 2 dose reductions are allowed. At each dose reduction, 1 tablet of abiraterone acetate/placebo will be removed, eg, 4 →3 tablets, and 3 →2 tablets. Any return to protocol dose level (4 tablets) after dose reduction must follow documentation of adverse event resolution and a discussion with the sponsor. For management of hepatotoxicity refer to section 7.5.9.2.

For patients who develop Grade ≥ 3 toxicities including hypertension, hypokalaemia, oedema and other, non mineralocorticoid toxicities, treatment should be withheld and appropriate medical management should be instituted. Treatment with abiraterone acetate should not be reinitiated until symptoms of the toxicity have resolved to Grade 1 or baseline.

7.5.9.1 Management of Mineralocorticoid Excess

Abiraterone acetate should be used with caution in subjects with a history of cardiovascular disease. Abirateone acetate may cause hypertension, hypokalaemia and fluid retention as a consequence of increased mineralocorticoid levels resulting from CYP17 inhibition. Caution is required in treating patients whose underlying medical conditions might be compromised by increases in blood pressure, hypokalaemia, or fluid retention.

In patients with pre-existing hypokalaemia or those that develop hypokalaemia whilst being treated with abiraterone actetate, consider maintaining the patient's potassium level at ≥ 4.0 mM.

7.5.9.2 Management of Hepatotoxicity

For patients who develop hepatotoxicity during treatment (alanine aminotransferase [ALT] increases or aspartate aminotransferare [AST] increases above 5 times the upper limit of normal [ULN]), treatment should be withheld immediately. Re-treatment following return of liver function tests to the patient's baseline may be given at a reduced dose of 500 mg (two tablets) once daily. For patients being re-treated, serum transaminases should be monitored at a minimum of every two weeks for three months and monthly thereafter. If hepatotoxicity recurs at the reduced dose of 500 mg daily, treatment should be discontinued.

If patients develop severe hepatotoxicity (ALT or AST 20 times the upper limit of normal) anytime while on therapy, treatment should be discontinued and patients should not be re-treated.

7.6 Criteria for Discontinuation of Study Treatment

The primary efficacy endpoint is rPFS, as defined in Sections 4.1.1 and 10.6.1.Confirmed radiographic progression may be a reliable indicator of clinical benefit in patients with CRPC [29]. Patients should ordinarily be maintained on study treatment until confirmed radiographic progression. If the patient has radiographic progression but no unequivocal clinical progression and alternate treatment is not initiated, the patient may continue on study treatment, at the investigator's discretion. At this point abiraterone acetate will not be supplied as study medication since in Germany abiraterone acetate is registered for the treatment of asymptomatic and mildly symptomatic CRPC.

However, if patients have unequivocal clinical progression without radiographic progression, these patients are indicated for the current standard of care. Study treatment should be stopped and patients advised regarding available treatment options.

For this study, unequivocal clinical progression will be characterized as:

1. Cancer pain requiring initiation of chronic administration of opiate analgesia (oral opiate use for ≥3 weeks; parenteral opiate use for ≥7 days, as listed in Appendix 9

OR

2. Immediate need to initiate cytotoxic chemotherapy or the immediate need to have either radiation therapy or surgical intervention for complications due to tumour progression, even in the absence of radiographic evidence of disease progression.

OR

3. Deterioration in ECOG performance status to grade 3 or higher.

   Patients whose ECOG performance status decreases to grade 2 during the study should be assessed carefully for their need for docetaxel therapy.

When study treatment is discontinued due to unequivocal clinical progression, the investigator should obtain further imaging studies to assess for radiographic progression, including a confirmatory bone scan, as appropriate.

Study treatment will be continued on patients who have increasing PSA values in the absence of radiographic or unequivocal clinical progression. Although serial PSA's will be measured on this study, progression or change in PSA values is not considered a reliable measure of disease progression, and should not be used as an indication to discontinue study therapy.
At the time of discontinuation of study treatment, re-initiation of LHRH-therapy is mandatory and must be recorded in patients who were randomized to the abiraterone plus prednisone arm.

7.7 Withdrawal from Study

A patient may withdraw from study treatment phase for any reason.

- In this event, the reason(s) for withdrawal must be documented and clarification requested whether withdrawal of consent applies only to the Treatment Phase (i.e. patient has not withdrawn consent for data collection during the post-treatment Follow-up Phase) or to both the Treatment and Follow-up Phases. A patient's decision to take part in the study voluntary, and he may choose not to take part in the study or to stop taking part at anytime. If he chooses not to take part or to stop at anytime, it will not affect his future medical care

An investigator may withdraw a patient from the study Treatment Phase at any time based on clinical judgment or for any of the following reasons listed below. When possible all End of Study Treatment and Follow-up procedures should be conducted.

- Discontinuation of treatment criteria as defined in Section 7.6
- Dosing noncompliance: Study treatment administration and dosing compliance will be assessed on Cycle 1 Day 15 visit. A count of study treatment will be conducted during this visit and the patient dosing compliance will be assessed. If dosing compliance is ≤75% in the absence of toxicity, patient should be re-instructed regarding proper dosing procedures and continue in the protocol. Subsequent dosing compliance procedure will be conducted at each visit. If a patient misses 14 or more doses within a single 28-day cycle, the patient should be discontinued from the study treatment Phase. All End-of-Study treatment procedures should be followed.
- Sustained Side Effects: Patients who have sustained toxicities, such as hyperglycemia or hypertension that do not return to NCI CTCAE (version 4) Grade 1 or less with appropriate medical management, should be discontinued from the study treatment Phase. All End-of-Study treatment procedures should be conducted. The patient will be followed until evidence of radiographic progression.
- Initiation of a new anticancer treatment: Patients will be discontinued from the protocol treatment when investigator determines new treatment for prostate cancer is warranted.The concurrent administration of other anticancer therapy, including cytotoxic, hormonal (except LHRH therapy in the appropriate treatment arm), or immunotherapy is prohibited during study treatment Phase. Use of other investigational drug therapy for any reason is prohibited.

8 Study Activities

8.1 Baseline (Day -28 to Day 0)

All patients must sign a written informed consent form before study specific screening procedures are performed. Screening procedures to evaluate patient eligibility for the study will be conducted within 28 days prior to Cycle 1 Day 1. If the patient meets eligibility and screening requirements he will be randomized and will return to the site for the Cycle 1 Day 1 visit and dosing.

The following activities/procedures will be conducted during the screening period, which may occur over 28 days:

- Medical history including prior prostate cancer therapies, PSA, Stage, and Gleason score at diagnosis
    - Previous hormonal, cytotoxic, and experimental treatments with start and stop dates
- Demographics
- Worst pain within last 24-hours
- Physical examination, weight, and height
- 12 lead ECG and an Echocardiogram (ECHO) – only if clinically indicated, i.e. in patients with known or suspicion of congestive heart failure (heart failure, uncontrolled hypertension, and history of myocardial infarction).
- Vital signs including upright blood pressure, heart rate, respiratory rate, and oral or aural body temperature
- Assessment of ECOG Performance Status

- Laboratory tests:
    - CBC: WBC with differential count, RBC, hemoglobin, hematocrit, platelets, neutrophiles
    - Chemistry with electrolytes: sodium, potassium, creatinine, BUN, albumin, total protein, calcium, glucose, uric acid, magnesium, chloride, albumin
    - Serum Lipids: Cholesterol, HDL, LDL, Triglycerides
- Liver function: ALK-P, ALT (SGPT), AST (SGOT), GGT, LDH, and total bilirubin Baseline tumour assessment:
    - CT or MRI of chest, abdomen and pelvis must be included. (the same method used at baseline should be used throughout the study) Tumour burden must be evaluated by physical examination and image-based evaluation (modified RECIST criteria 1.1).Ultrasound should not be used to measure lesions that are not clinically accessible, such as liver lesions. The method used for baseline tumour assessment should be used for all subsequent tumour assessment throughout the study treatment phase. Chest X-ray is not recommended. Scans performed up to 28 days prior to Cycle 1 Day 1 can be used for baseline assessments.
    - Bone Scan
        - Scans performed up to 28 days prior to Cycle 1 Day 1 can be used for baseline assessments.
    - Concomitant medications listing
        - Obtain a complete and thorough listing of all prescription and nonprescription (over the counter) medications currently taken including pain medications. This also includes any nutritional supplements and/or herbal preparations.

8.2 Randomization

Once eligibility is confirmed AND written informed consent is signed by patient, patients will be randomized to a treatment group according to the randomization schedule. All patients must commence treatment (Cycle 1 Day 1) within 72 hours (3 calendar days) of randomization.

8.3 Treatment Period

Randomized patients will have Cycle 1 Day 1 procedures and receive study treatment (abiraterone acetate) that will subsequently be administered orally once daily until discontinuation of study treatment. After discontinuation abiraterone acetate may be continued on discretion of the treating physician. All patients will also take 5mg of prednisone or prednisolone orally twice daily. If a patient has been receiving glucocorticoids other than prednisone or prednisolone, it will be necessary to switch the glucocorticoid to prednisone or prednisolone 5mg bid prior to Cycle 1 Day 1. Patients who are randomized to the abiraterone + prednisone + LHRH-therapy arm will continue on the same LHRH-therapy that had been used prior to randomization. Patients who are randomized to the abiraterone + prednisone arm will not continue on LHRH-therapy from Cycle 1 Day 1 until discontinuation of study treatment. These patients may resume on LHRH-therapy after study discontinuation on the discretion of the treating physician.

No crossover will be permitted between the 2 treatment groups. Each cycle of treatment will be 28 days. Patients will return for a Cycle 1 Day 15 visit ± 2 days to evaluate safety and dosing adherence (a count of study drug tablets). From Cycle 2 to End of Study Treatment, Day 1 visits will occur every 28 days with a ± 3 days window. Study windows are to be calculated from Cycle 1 Day 1 date, and if utilized, every effort will be made for the patient to return to schedule. Patients

may have additional imaging visits up to 8 days before Cycles requiring images (Cycle 4 Day 1, Cycle 7 Day 1, and Cycle 10 Day 1 and every 3rd Cycle beyond cycle 10) or at Treatment Discontinuation Visit.

8.3.1 Cycle 1 Day 1

Patients who are eligible will be randomized and start study treatment within 28 days after the screening visit.

Cycle 1 Day 1 visit may occur on the same day as the Screening visit provided that all screening assessments have been completed and screening results are reviewed prior to the commencement of Cycle 1 Day 1 assessments.

The following procedures should be carried out prior to dosing of study treatment:

- Update listing of current baseline signs and symptoms with associated NCI Common Terminology Criteria for Adverse Events grading (0-4) with any event that may have occurred since screening
- Worst pain within last 24-hours
- Concomitant Medications listing
- Vital signs including upright blood pressure, heart rate, respiratory rate, and oral or aural body temperature
- Assessment of ECOG Performance Status
- Laboratory tests
    - PSA: If patient undergoes a DRE, PSA must be sampled prior to DRE
    - ALK-P, LDH
    - Blood specimen for hormone analysis (central lab): Serum testosterone, DHEA-S, Luteinizing Hormone (LH), Luteinizing Hormone Releasing Hormone (LHRH), Follicle Stimulating Hormone (FSH), and Dihydrotestosterone (DHT)

8.3.2 Cycle 1 Day 15 Visit

The following assessments should be carried out at 14 days post-dose visit on Day 15:

- Worst pain within last 24-hours
- Physical examination and weight
- Vital signs including upright blood pressure and heart rate
- Assessment of ECOG Performance Status
- Laboratory tests
    - Chemistry with electrolytes: sodium, potassium, creatinine, BUN, albumin, total protein, calcium, glucose, uric acid, magnesium, chloride. albumin
    - Liver function: ALK-P, ALT (SGPT), AST (SGOT), GGT, LDH, and total bilirubin.
- Concomitant Medications listing
- AE evaluation and recording will be monitored throughout the study. At each post baseline visit, the investigator will begin by querying for adverse events by asking each patient a

general, non-directed question such as "How have you been feeling since the last visit?" Directed questioning and examination will then be done as appropriate.

- Dosing compliance check, a count of study treatment tablets, will be conducted during this visit and patient dosing compliance will be assessed. If dosing compliance is ≤75% in the absence of toxicity, patient should be reinstructed regarding proper dosing procedures and may continue in the protocol. Source documentation of the compliance check and any patient verbal re-instruction provided should be documented in patient's chart.

8.3.3 Cycle 2 and 3 Day 1

Within this study, Data on safety will be collected every 4 weeks from cycle 2 on. However, the treating physician should be aware that liver enzymes (i.e. AST, ALT, ALK-P) should be measured according to the current "Fachinformation".

- Worst pain within last 24-hours
- Physical examination and weight
- Vital signs including upright blood pressure and heart rate
- Assessment of ECOG Performance Status
- AE evaluation and recording
- Blood specimen for hormone analysis (central lab): Serum testosterone, DHEA-S, Luteinizing Hormone (LH), Luteinizing Hormone Releasing Hormone (LHRH), Follicle Stimulating Hormone (FSH), and Dihydrotestosterone (DHT)
- Laboratory tests
    - CBC: WBC, RBC, hemoglobin, hematocrit, platelets, neutrophiles
    - Chemistry with electrolytes: sodium, potassium, creatinine, BUN, albumin, total protein, calcium, glucose, uric acid, magnesium, chloride, albuminSerum Lipids: Cholesterol, HDL, LDL, TriglyceridesLiver function: ALK-P, ALT (SGPT), AST (SGOT), GGT, albumin, LDH, and total bilirubin.
    - Liver function: ALK-P, ALT (SGPT), AST (SGOT), GGT, LDH, and total bilirubin.
    - Only at Cycle 2+3 Day 1: Blood specimen for hormone analysis (central lab) including Serum testosterone, DHEA-S, Luteinizing Hormone (LH), Luteinizing Hormone Releasing Hormone (LHRH), Follicle Stimulating Hormone (FSH), and Dihydrotestosterone (DHT)
    - PSA: If patient undergoes a DRE, then PSA must be samples prior to the DRE
- Concomitant Medications listing
- Dosing compliance check, a count of study treatment tablets, will be conducted during this visit and patients dosing compliance will be assessed. If compliance is ≤75% patient should be re-instructed regarding proper dosing procedures. Patients whose dosing compliance is ≤75% for 2 consecutive cycles should be discontinued from the study. Patients who have a compliance ≤75% secondary to held doses due to toxicities as described in Section7.5.9 may continue in the study. Source documentation of the compliance check and any patient verbal re-instruction provided should be documented in patient's chart.

8.3.4 Cycle 4 Day 1 and any other following cycles

Within this study, Data on safety will be collected every 4 weeks from cycle 2 on. However, the treating physician should be aware that liver enzymes (i.e. AST, ALT, ALK-P) should be measured according to the current "Fachinformation".

- Worst pain within last 24-hours
- Physical examination and weight
- Vital signs including upright blood pressure and heart rate
- Assessment of ECOG Performance Status
- AE evaluation and recording
- Laboratory tests
    - o CBC: WBC, RBC, hemoglobin, hematocrit, platelets, neutrophiles
    - o Chemistry with electrolytes: sodium, potassium, creatinine, BUN, albumin, total protein, calcium, glucose, uric acid, magnesium, chloride, albumin
    - o Liver function: ALK-P, ALT (SGPT), AST (SGOT), GGT, LDH and total bilirubin.
    - o PSA: If patient undergoes a DRE, then PSA must be samples prior to the DRE
- Concomitant Medications listing
- Dosing compliance check, a count of study treatment tablets, will be conducted during this visit and patients dosing compliance will be assessed. If compliance is ≤75% patient should be re-instructed regarding proper dosing procedures. Patients whose dosing compliance is ≤75% for 2 consecutive cycles should be discontinued from the study. Patients who have a compliance ≤75% secondary to held doses due to toxicities as described in Section7.5.9 may continue in the study. Source documentation of the compliance check and any patient verbal re-instruction provided should be documented in patient's chart.

8.3.5 Cycles 4, 7, and 10 Day 1 (continue every 3rd cycle beyond Cycle 10, e.g. Cycles 13, 16, 19, 22...)

At these visits, perform assessments as outlined in 8.3.3. Additionally, the following assessments should be conducted up to 8 days prior to the indicated visit (imaging procedures) or at the day of visit (hormone analysis).

- CT or MRI (the same method used at baseline should be used throughout the study)
- Bone Scan: If progressive disease is observed on bone scan at any timepoint (without RECIST progression on CT/MRT), a confimatory bone scan is required at least six weeks later. The patient should remain on study treatment in the interim unless there is unequivocal clinical progression as defined in section 7.6.
- Blood specimen for hormone analysis (central lab): Serum testosterone, DHEA-S, Luteinizing Hormone (LH), Luteinizing Hormone Releasing Hormone (LHRH), Follicle Stimulating Hormone (FSH), and Dihydrotestosterone (DHT)
- ECG and ECHO should be repeated if clinically indicated, i.e. in patients with known or suspicion of congestive heart failure (heart failure, uncontrolled hypertension, history of myocardial infarction) or symptoms and signs of heart failure.

8.3.6 Treatment Discontinuation Visit

The Treatment Discontinuation Visit can occur at any scheduled or unscheduled visit when applicable. At this visit, documentation to confirm progressive disease or unequivocal clinical progression as defined in Section 7.6is required.

- Blood specimen for hormone analysis (central lab): Serum testosterone, DHEA-S, Luteinizing Hormone (LH), Luteinizing Hormone Releasing Hormone (LHRH), Follicle Stimulating Hormone (FSH), and Dihydrotestosterone (DHT)

8.3.7 End-of-Treatment Visit

The following safety assessments should be carried out between 15 to 29 days after last dosing of study treatment:

- Physical examination and weight
- Worst pain within last 24-hours
- Vital signs including upright blood pressure and heart rate
- Assessment of ECOG Performance Status
- Laboratory tests
    - o CBC: WBC, RBC, hemoglobin, hematocrit, platelets
    - o Chemistry with electrolytes: sodium, potassium, creatinine, BUN, albumin, total protein, calcium, glucose, uric acid, magnesium
    - o Liver function: ALK-P, ALT (SGPT), AST (SGOT), LDH, and total bilirubin.
    - o Blood specimen for hormone analysis (central lab): Serum testosterone, DHEA-S, Luteinizing Hormone (LH), Luteinizing Hormone Releasing Hormone (LHRH), Follicle Stimulating Hormone (FSH), and Dihydrotestosterone (DHT)
    - o PSA: If patient undergoes a DRE, then PSA must be samples prior to the DRE
- AE evaluation and recording
- Concomitant Medications listing
- Final Dosing compliance check

8.3.8 Post-Treatment Follow-up Period

Follow-up for each patient will continue until radiographic progression occurs, patient dies, is lost to follow-up, withdraws informed consent or until end of the study, whichever occurs earlier. Follow-up for all patients ends when rPFS at month 12 had been determined for the last patient on study treatment.

Follow-up contacts should be assessed every 3 months until radiographic progression, death, lost to follow-up, withdrawal of informed consent, or until end of the study, whichever occurs earlier. Follow-up for all patients ends when rPFS at month 12 can be measured for the last patient on study treatment.

The following assessments should be carried out during follow up when patients are off study treatment:

- CT, MRI, or other imaging procedures as clinically indicated and as defined in Appendix 4

- Bone Scan

  If progressive disease is observed on bone scan at any time (without RECIST progression on CT/MRT), a confirmatory bone scan is required at least six weeks later. The patient should remain on study treatment in the interim unless there is unequivocal clinical progression as defined in section 7.6.

- PSA: If patient undergoes a DRE, then PSA must be samples prior to the DRE
- Next therapy for prostate cancer
- Serious and non-serious adverse events (SAEs and AEs) considered at least possibly related to investigationaldrug will be collected and reported to the sponsor within 24-hours of discovery or notification of the event.
- For patients who discontinue treatment for reasons other than radiographic progression tumour assessments should continue until confirmed radiographic progression.

9 Quality Control and Assurance

During and/or after completion of the study, regulatory authorities may wish to perform on-site audits. The investigators will be expected to cooperate with any audit and to provide assistance and documentation (including source data) as requested.

In accordance with ICH CGP this study may be selected for audit by regulatory authorities. Inspection of site facilities (e.g., pharmacy, drug storage areas, laboratories) and review of study-related records will occur to evaluate the study conduct and compliance with the protocol, ICH Good Clinical Practice (ICH E6), EU Clinical Trials Directive (Directive 2001/20/EC), and applicable regional regulatory requirements.

Apart from the regulatory authorities, the sponsor may also wish to perform on-site audits. In this case the same cooperation as outlined above will be expected.

The Sponsor representatives (Monitors) are responsible for contacting and visiting the investigator for the purpose of inspecting the facilities and, upon request, inspecting the various records of the clinical study (e.g., CRFs and other pertinent data) provided that patient confidentiality is respected.

The investigator agrees to cooperate with the monitor to ensure that any problems detected in the course of these monitoring visits, including delays in completing CRFs, are resolved.

10 Planned Statistical Methods

10.1 General considerations

All statistical analyses will be performed using SAS 9.3 (or higher). The resulting statistic will be evaluated using the Chi-square test as implemented in the SAS procedure FREQ given the precise number of events observed until the time of interest. Unless otherwise specified, all continuous endpoints will be summarized using descriptive statistics, which will include the number of patients (n), mean, standard deviation, median, minimum, and maximum. All categorical endpoints will be summarized using frequencies and percentages. The baseline measurement will be the last value on or before the date of first study treatment.

10.2 Determination of Sample Size

Primary analysis for radiographic progression-free survival will be based on the full analysis set. In a controlled phase III-trial (COU-AA-302) radiographic progression-free survival was 16.5 vs. 8.3 months in the abiraterone arm and the placebo arm, respectively[2]:

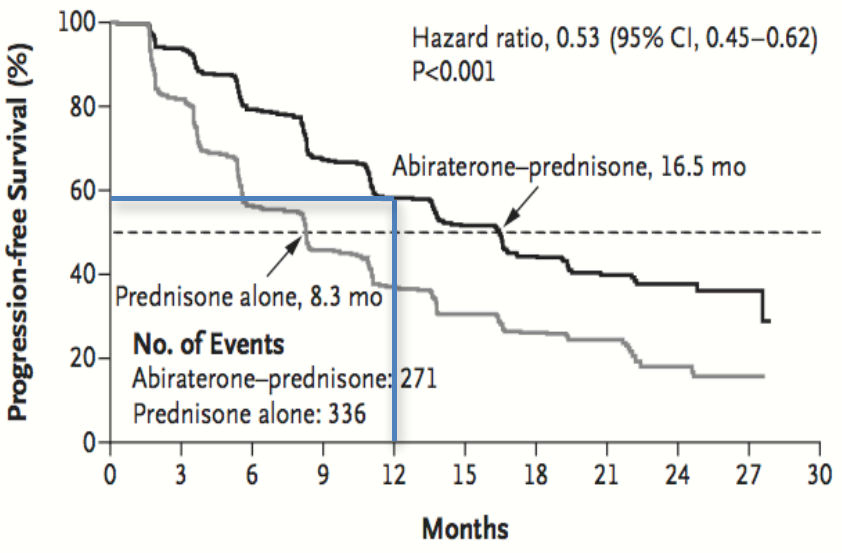

Figure 10.1 Progression-free survival in the COU-AA-302 trial [2].

For this trial, the primary efficacy parameter will be the rate of radiographic progression-free survival after 12 months.

Since this is an exploratory phase-II trial neither the primary endpoint radiographic progression-free survival (rPFS) nor the secondary endpoints will be powered for statistical significance. Each treatment arm will consist of 30 patients evaluable for the primary endpoint rPFS. Assuming a drop-out rate of 15% in each arm it is estimated that 70 patients need to be recruited for this trial at 12 sites across Germany. Based on the interim results from the COU-AA-302 trial the median time on treatment within this trial is estimated to be 15 months [32]. Accrual time for the trial is estimated to take 12 months.

The results of the trial may give way for a randomized phase III trial comparing AA + Pred + LHRH-therapy versus AA + Pred if a clinical relevant difference is suspected based on the results of this trial.

10.3 Analysis Populations

Patient disposition and efficacy analyses will be performed on data from the intention-to-treat (ITT) population. All patients randomized into the study will be classified according to their assigned treatment group, regardless of the actual treatment received. The primary efficacy analyses will be on the ITT basis.

All patients who receive any part of abiraterone acetate will be included in the analysis of safety (Safety Population)

10.4 Interim Analysis

An interim analysis is not planned for the trial.

10.5 Demographics and Baseline Characteristics

Demographic variables will include age, height, and weight. Baseline disease characteristics will include time from diagnosis, number of prior hormonal therapies, time from initiation of primary hormone therapy to start of abiraterone and other clinical characteristics as documented in the CRF.

10.6 Study endpoint(s)

10.6.1 Efficacy Endpoint(s)

Primary efficacy endpoint:

- Radiographic progression-free survival (rPFS) as rate of progression free survival after 12 months

Secondary efficacy endpoints:

- PSA response rate scored in patients achieving a post-treatment PSA decline of at least 50% according to the protocol-specific PCWG2 criteria
- Time to PSA-progression will be measured from the time interval from the date of randomization to the date of the PSA progression as defined in the protocol-specific PCWG2 criteria. The determination of PSA progression will require that the patient receive at least 3 cycles of therapy.
- Objective response rate in patients with measurable disease (RECIST)
- Value of the bone-scan index as a biomarker of response to treatment
- Changes in pituitary gonadal axis by measurement of androgens and hormones (LHRH, LH, FSH, testosterone, DHT)
- Safety

10.7 Efficacy Analysis Population and Methods

Radiographic progression-free survival is defined as the time from randomization to the occurrence of radiographic progression or death (Section 4.1.1). The analysis of rPFS endpoint will use the investigator radiographic assessment of progression.

10.8 Safety Evaluations

Safety analysis will be summarized using the Safety Population.

Extent of exposure to study drug will be summarized and details will be provided.

Treatment emergent adverse events (AEs) are those events that occur or worsen on or after first dose of study drug up through 30 days post last dose. Adverse events will be coded using the MedDRA coding system and all AEs will be graded according to the National Cancer Institute Common Terminology Criteria for Adverse Events, version 4.0 (NCI- CTCAE).

Incidence of AEs will be summarized by system organ class (SOC) and preferred term (PT), and will be presented by treatment groups and overall. Adverse events will be summarized by grade, according to the worst grade experienced. In addition, most frequently observed adverse events will be summarized by treatment groups. In the summary of AE, an AE occurs more than once within a SOC and PT will be counted only once using the worst grade experienced.

Serious AE and deaths will be provided in a listing. All adverse events resulting in discontinuation, dose modification, dosing interruption, and/or treatment delay of study drug will also be listed and tabulated by preferred term.

Clinical laboratory test results will be collected pretreatment and through 30 days post last dose of study treatment. All laboratory test results will be classified according to the NCI CTCAE v4.0 criteria. Standard reference ranges will be used for missing or discrepant normal ranges. Baseline laboratory test values are the results from the last blood samples drawn on or prior to the first day of study treatment. On-study laboratory test values are those results from blood samples drawn after the first study treatment up until 30 days after the last dose of study treatment.

Mean change from baseline in laboratory test values at each visit will be provided. On-study clinical laboratory test abnormalities will be summarized. Shifts in laboratory test values will also be summarized.

11 Steering committee

The study is accompanied by a study steering committee. The members are assigned by the sponsor and include the trial coordinator, 1 representative of the sponsor and 1 Janssen-Cilag representative. The Janssen-Cilag representative is providing information and advice with regard to Abiraterone, but has no voting rights with respect to any study procedures. The sponsor chairs the committee. The steering committee will evaluate the conduct of this trial and assesses the study progress and safety (including interim data); the members having voting rights will make any necessary recommendations, as needed, an will also develop study-related publications.

12 Data Monitoring Committee

An independent Data Monitoring Committee (DMC) is not planned for the trial. According to the guidelines on data monitoring commiettees of the European Medical Association (EMA) the steering committee regard the potential harm of the patient to be minimal (see also 3) so that a DMC was considered not to be needed for this trial.

13 Compensation, Insurance, and Indemnity

In accordance with the provisions of the German Drug Law (AMG) and ICH-GCP, the sponsor has an insurance policy intended to guarantee against possible damages resulting from research. The studies and/or experiments performed on behalf of the sponsor are specifically and expressly guaranteed. It is advisable to underline that non-compliance with the research legal conditions are clauses of guarantee of exclusion.

14 Administrative responsibilities

14.1 Investigators and Study Administrative Structure

14.1.1 Investigator Responsibilities

The investigator undertakes to perform the study in accordance with ICH Guidelines per Good Clinical Practice (GCP) and the applicable regulatory requirements. A copy of the guidelines will be available in the Investigator Site File.

It is the investigator’s responsibility to ensure that adequate time and appropriate resources are available at the investigational site prior to commitment to participate in this study. The investigator should also be able to demonstrate a potential for recruiting the required number of suitable patients within the agreed recruitment period.

The investigator will maintain a list of appropriately qualified persons to whom the investigator has delegated significant study-related tasks. An up-to-date copy of the curriculum vitae for the investigators will be provided to the sponsor (or its representatives) before starting the study.

If the patient has a primary physician, the investigator should, with the patient’s consent, inform them of the patient’s participation in the study.

14.1.2 Protocol Adherence and Investigator Agreement

The investigator must adhere to the protocol as detailed in this document. The investigator will be responsible for enrolling only those patients who have met protocol eligibility criteria. The investigator will be required to sign an investigator agreement to confirm acceptance and willingness to comply with the study protocol.

It is the sponsor’s responsibility to communicate with their localEC to ensure accurate and timely information is provided at all phases during the study. In particular, the appropriate approvals must

be in place prior to recruitment, notification of any SAEs during the study must take place and the EC must be informed of study completion.

14.2 Independent Ethics Committee (EC) Approval

It is the responsibility of the sponsor to submit this protocol, the final approved informed consent document (approved by the sponsor or its representative), relevant supporting information, and all types of patient recruitment or advertisement information (approved by the sponsor or its representative) to the EC for review and it must be approved before the study is initiated. Prior to implementing changes in the study, the sponsor will produce a protocol amendment and the EC must also approve any amendments to the protocol.

Drug supplies will not be released and the patient recruitment will not begin until this written approval has been received by the sponsor or its designee.

The investigator is responsible for keeping theEC apprised of the progress of the study and of any changes made to the protocol as deemed appropriate. The investigator must also keep the EC informed of any serious and significant adverse events.

14.3 Ethical Conduct of the Study

This protocol accords with the principles of the Declaration of Helsinki as set forth at the 18th World Medicines Association (Helsinki 1964) and amendments of the 29th (Tokyo 1975), the 35th (Venice 1983), the 41st (Hong Kong 1989), the 48th, and the 52nd (Edinburgh) World Medicines Association. As these accords are reviewed and amended periodically, the most current amended version will be in effect.

14.4 Patient Information and Consent

It is the responsibility of the investigator to obtain written informed consent from patients. Each patient is requested to sign the patient Information and Consent Form after the patient has received written information and an explanation of what the study involves, i.e., the objectives, potential benefits and risk, inconveniences and the patient's rights and responsibilities. A copy of the Patient Information and signed Consent Form must be given to the patient. The Patient Information and Consent Form will be provided in German. Signed consent forms must be available for verification by study monitors at any time.

14.5 Patient Confidentiality

Data collected during this study may be used to support the development, registration or marketing of abiraterone acetate. All data collected during the study will be controlled by the sponsor or designee and the sponsor will abide by all relevant data protection laws. After patients have consented to take part in the study, their medical records and the data collected during the study will be reviewed by the sponsor or its designee to confirm that the data collected are accurate and for the purpose of analyzing the results. These records and data may additionally be reviewed by auditors or by regulatory authorities. The patient's name, however, will not be disclosed outside the hospital. Each patient will be pseudonymized by a unique patient number. The results of this study may be used in other countries throughout the world that have ensured an adequate level of protection for personal data.

Data Protection Consent is to be obtained from each patient prior to enrollment into the study in accordance with the applicable privacy requirements defined by the German data protection laws.

14.6 Study Monitoring

To ensure compliance with relevant regulations, data generated by this study must be available for inspection upon request by representatives of the EMEA, the BfArM, and local health authorities, the sponsor and his representatives, and theEC for each study site. The sponsor of the study may delegate some administrative aspects of the study to a duly authorized representative including, but not limited to, study initiation, monitoring, and management of SAE reports.

The sponsor or representative's monitor is responsible for verifying the eCRFs at regular intervals throughout the study to verify adherence to the protocol; completeness, accuracy, and consistency of the data; and adherence to local regulations on the conduct of clinical research. The monitor should have access to the patients' medical records and other study-related records needed to verify the entries on the eCRFs.

The monitor (auditors, EC or regulatory inspectors) will check the CRF entries against the source documents. The consent form will include a statement by which the patients allow the monitor/auditor/inspector from the EC or regulatory authority access to the source data (eg, patient's medical file, appointment books, original laboratory test reports, X rays etc) that substantiate information in the CRFs. These personnel, bound by professional secrecy, will not disclose any personal information or personal drug information.

14.7 Case Report Forms

Electronic Case Report Forms (eCRFs) will be provided by the sponsor or its representatives and should be handled in accordance with instructions from the sponsor or its representative.

The investigator is responsible for maintaining adequate and accurate eCRFs which have been designed to record all observations and other data pertinent to the clinical investigation. Each eCRF should be filled out completely by the investigator or delegate as stated in the Site Delegation List. All data captured for the study is planned to be electronic. However, if necessary, data capture may be performed using paper CRFs.

If paper CRFs are used, then all CRFs should be completed in a neat legible manner to ensure accurate interpretation of the data; a black all-point pen should be used to ensure the clarity of reproduced copies of all CRFs. Incorrect entries should be crossed with a single line. Corrections must be made adjacent to the item to be altered, initialed and dated with the reason for the correction if necessary, by an authorized (investigator/co-worker) person. Overwriting of this information or use of liquid correction fluid is not allowed.

The eCRFs should be reviewed, signed and dated by the investigator.

Once the site monitor has verified the contents of the completed eCRF pages against the source data, the EDC system will be locked for those pages. Queries may be raised if the data are unclear or contradictory, which must be addressed by the investigator.

14.8 Laboratory Assessments

Local and one central laboratory will be responsible to analyze safety labs collected for the study as well as the additional and specialty lab tests. All local laboratory results will be captured on supplemental lab eCRFs.

14.9 Radiologic Review

In this study, all CT, MRI, and Bone scans will be analyzed by local radiological and/or nuclear medicine department using modified RECIST criteria (Version 1.1). Central collection and retrospective independent review of radiographic progression will not be performed. For the

determination of the bone-scan index, digitalized bone scans will be collected and analyzed centrally.

14.10 Protocol Violations/Deviations

Protocol deviations should be reported to the sponsor (or designee) as they occur or are discovered and should be captured in eCRFs at the time of monitoring and medical review of data line listings.

14.11 Access to Source Documentation

Source data to be reviewed during this study will include, but is not restricted to: patient's medical file, patient's diary cards (if applicable), and original laboratory test, histology, and pathology reports. All key data must be recorded in the patient's hospital notes.

14.12 Retention of Data

As described in the ICH GCP Guidelines, 'essential documents', including CRFs, source documents, consent forms, laboratory test results, and drug inventory records, should be retained by the investigator until at least 2 years after the last approval of a marketing application in an ICH region and until there are no pending or contemplated marketing application in an ICH region or at least 2 years have elapsed since the formal discontinuation of clinical development of the investigational product. These documents should be retained for a longer period however if required by the applicable regulatory requirements or by an agreement with the sponsor. The investigator should obtain written permission by the sponsor prior to destruction of any study document.

These records should be made available at reasonable times for inspection and duplication, if required, by a properly authorized representative of the National Regulatory Authorities.

14.13 Financial Disclosure

The investigator will be required to disclose any financial arrangement whereby the value of the compensation for conducting the study could be influenced by the outcome of the study following information; any significant payments of other sorts from Johnson&Johnson or Janssen-Cilag GmbH, such as a grant to fund ongoing research, compensation in the form of equipment, retainer for ongoing consultation, or honoraria; any proprietary interest in abiraterone acetate; any significant equity interest in Johnson&Johnson or Janssen-Cilag GmbH as defined in the US Code of Federal Regulations (21 CFR 54 2(b)).

In consideration of participation in the study, the sponsor will pay the investigator, or nominated payee the sums set out in the payment schedule attached to the Investigator Agreement.

14.14 Study Publication Guidelines and Disclosures Policy

The sponsor encourages publication of results derived from the clinical research it sponsors. Publication includes publication of a paper in a peer-reviewed medical journal, abstract submission with a poster or oral presentation at a scientific meeting, or making results public by some other means. The sponsor will retain the right to review all material prior to presentation or submission for publication and neither institution(s) nor Principal Investigator(s) is/are permitted to publish/present the results of the study, in part or in the entirely without the written authorization of Sponsor.
First publication

The results of the entire multi-center study shall be presented in a first publication upon completion of the entire multi-center study (data lock), with authorship being determined by the sponsor using

the criteria defined by the International Committee of Medical Journal Editors. The sponsor will be included as the first author on the first publication of the results.

Subsequent Publications

Results from data subsets should not be published in advance of and must make reference to the first publication. Publications must include the sponsor to allow recognition of the Sponsor's involvement.

In all publications, the study is to be identified as SPARE-001. The study Co-Chairs/Principal Investigator(s) shall be free to publish or present, subject to the timing described in the Clinical Trial Agreement.

References

1. Scher HI, Heller G, Molina A, et al. Evaluation of circulating tumor cell (CTC) enumeration as an efficacy response biomarker of overall survival (OS) in metastatic castration-resistant prostate cancer (mCRPC): Planned final analysis (FA) of COU-AA-301, a randomized, double-blind, placebo-controlled, phase III study of abiraterone acetate (AA) plus low-dose prednisone (P) post docetaxel. ASCO Meeting Abstracts 2011;29:LBA4517
2. Ryan CJ, Smith MR, de Bono JS, et al. Abiraterone in metastatic prostate cancer without previous chemotherapy. N Engl J Med 2012;368:138-148
3. Ryan CJ, Smith MR, Fong L, et al. Phase I clinical trial of the CYP17 inhibitor abiraterone acetate demonstrating clinical activity in patients with castration-resistant prostate cancer who received prior ketoconazole therapy. J Clin Oncol 2010;28:1481-1488
4. Mottet N, Bellmunt J, Bolla M, et al. EAU guidelines on prostate cancer. Part II: Treatment of advanced, relapsing, and castration-resistant prostate cancer. Eur Urol;59:572-583
5. Kobayashi T, Nishizawa K, Mitsumori K. Individual variation of hormonal recovery after cessation of luteinizing hormone-releasing hormone agonist therapy in men receiving long-term medical castration therapy for prostate cancer. Scand J Urol Nephrol 2006;40:198-203
6. Kaku H, Saika T, Tsushima T, et al. Time course of serum testosterone and luteinizing hormone levels after cessation of long-term luteinizing hormone-releasing hormone agonist treatment in patients with prostate cancer. Prostate 2006;66:439-444
7. Barrie SE, Potter GA, Goddard PM, et al. Pharmacology of novel steroidal inhibitors of cytochrome P450(17) alpha (17 alpha-hydroxylase/C17-20 lyase). J Steroid Biochem Mol Biol 1994;50:267-273
8. O'Donnell A, Judson I, Dowsett M, et al. Hormonal impact of the 17alpha-hydroxylase/C(17,20)-lyase inhibitor abiraterone acetate (CB7630) in patients with prostate cancer. Br J Cancer 2004;90:2317-2325
9. Pinski J, Xiong S, Wang Q, et al. Effect of luteinizing hormone on the steroidogenic pathway in prostate cancer. Prostate 2006;71:892-898
10. Mostaghel EA, Marck BT, Plymate SR, et al. Resistance to CYP17A1 inhibition with abiraterone in castration-resistant prostate cancer: induction of steroidogenesis and androgen receptor splice variants. Clin Cancer Res;17:5913-5925
11. Mohler JL, Titus MA, Wilson EM. Potential prostate cancer drug target: Bioactivation of androstanediol by conversion to dihydrotestosterone. Clin Cancer Res 2011;ePub
12. Ryan CJ, Smith MR, de Bono JS, et al. Abiraterone in metastatic prostate cancer without previous chemotherapy. N Engl J Med 2013;368:138-148
13. Reid AH, Attard G, Danila DC, et al. Significant and sustained antitumor activity in post-docetaxel, castration-resistant prostate cancer with the CYP17 inhibitor abiraterone acetate. J Clin Oncol;28:1489-1495
14. de Bono JS, Logothetis CJ, Molina A, et al. Abiraterone and increased survival in metastatic prostate cancer. N Engl J Med 2011;364:1995-2005
15. Attard G, Reid AH, Yap TA, et al. Phase I clinical trial of a selective inhibitor of CYP17, abiraterone acetate, confirms that castration-resistant prostate cancer commonly remains hormone driven. J Clin Oncol 2008;26:4563-4571
16. Auchus RJ. The genetics, pathophysiology, and management of human deficiencies of P450c17. Endocrinol Metab Clin North Am 2001;30:101-119, vii
17. de Bono JS, Attard G, Adjei A, et al. Potential applications for circulating tumor cells expressing the insulin-like growth factor-I receptor. Clin Cancer Res 2007;13:3611-3616

18. Attard G, Reid A, Sinha R, et al. Selective inhibition of CYP17 with abiraterone acetate is well tolerated and results in a high response rate in castration-resistant prostate cancer (CRPC). AACR Meeting Abstracts 2007;2007:B73
19. Attard G, Reid AH, A'Hern R, et al. Selective inhibition of CYP17 with abiraterone acetate is highly active in the treatment of castration-resistant prostate cancer. J Clin Oncol 2009;27:3742-3748
20. Fizazi K, Scher HI, Molina A, et al. Abiraterone acetate for treatment of metastatic castration-resistant prostate cancer: final overall survival analysis of the COU-AA-301 randomised, double-blind, placebo-controlled phase 3 study. Lancet Oncol 2012;13:983-992
21. Scher HI, Morris MJ, Kelly WK, Schwartz LH, Heller G. Prostate cancer clinical trial end points: "RECIST"ing a step backwards. Clin Cancer Res 2005;11:5223-5232
22. Pollen JJ, Witztum KF, Ashburn WL. The flare phenomenon on radionuclide bone scan in metastatic prostate cancer. AJR Am J Roentgenol 1984;142:773-776
23. Scher HI, Warren M, Heller G. The association between measures of progression and survival in castrate-metastatic prostate cancer. Clin Cancer Res 2007;13:1488-1492
24. Bubley GJ, Carducci M, Dahut W, et al. Eligibility and response guidelines for phase II clinical trials in androgen-independent prostate cancer: recommendations from the Prostate-Specific Antigen Working Group. J Clin Oncol 1999;17:3461-3467
25. Halabi S, Vogelzang NJ, Ou SS, et al. Progression-free survival as a predictor of overall survival in men with castrate-resistant prostate cancer. J Clin Oncol 2009;27:2766-2771
26. Armstrong AJ, Eisenberger MA, Halabi S, et al. Biomarkers in the management and treatment of men with metastatic castration-resistant prostate cancer. Eur Urol 2012;61:549-559
27. Carducci MA, Saad F, Abrahamsson PA, et al. A phase 3 randomized controlled trial of the efficacy and safety of atrasentan in men with metastatic hormone-refractory prostate cancer. Cancer 2007;110:1959-1966
28. Brundage MD, Crook JM, Lukka H. Use of strontium-89 in endocrine-refractory prostate cancer metastatic to bone. Provincial Genitourinary Cancer Disease Site Group. Cancer Prev Control 1998;2:79-87
29. Scher HI, Halabi S, Tannock I, et al. Design and end points of clinical trials for patients with progressive prostate cancer and castrate levels of testosterone: recommendations of the Prostate Cancer Clinical Trials Working Group. J Clin Oncol 2008;26:1148-1159
30. Scher HI, Morris MJ, Basch E, Heller G. End points and outcomes in castration-resistant prostate cancer: from clinical trials to clinical practice. J Clin Oncol 2011;29:3695-3704
31. Heidenreich A, Albers P, Classen J, et al. Imaging studies in metastatic urogenital cancer patients undergoing systemic therapy: recommendations of a multidisciplinary consensus meeting of the Association of Urological Oncology of the German Cancer Society. Urol Int;85:1-10
32. Ryan CJ. Interim analysis (IA) results of COU-AA-302, a randomized, phase III study of abiraterone acetate (AA) in chemotherapy-naive patients (pts) with metastatic castration-resistant prostate cancer (mCRPC). In: J Clin Oncol 30, 2012 (suppl; abstr LBA4518); 2012

15 Appendices

Appendix 1 **Schedule of Events**

Table of Scheduled Events

			Treatment Phase (cycle length = 28 days)						Follow-up Phase
Required Investigations	Baseline Day -28 to 0	Cycle 1 Day 1	Cycle 1 Day 15	[1]Cycle 2 and 3 Day 1,	Cycles 4, 7, 10 (continue every 3rd cycle) Day 1	Any other Cycle, Day 1	At Treatment Discontinuation[2, 18]	End-of-Treatment Visit[3]	Follow-up visit[6] (every 3 months)
**Procedures**									
Signed consent form[4]	X								
Medical history, prior Prostate cancer therapies	X								
Worst pain within last 24 hours	X	X	X	X	X	X		X	
Physical exam and Weight	X		X	X	X	X		X	
Vital signs[5]	X	X	X	X	X	X		X	
ECOG	X	X	X	X	X	X		X	
12 Lead ECG[6]	X				X[6]			X[6]	
Cardiac ECHO[6]	X				X[6]				
Dosing compliance			X	X	X	X		X	
Concomitant medication	X	X	X	X	X	X		X	
Adverse events	X[7]	X	X	X	X	X		X[8]	X
**Laboratory Assessments**									
Hematology	X			X	X	X		X	
Serum chemistry, electrolytes	X		X	X	X	X		X	
Liver function test	X[9]		X	X	X	X		X	
Serum Lipids	X		X	X	X				
Glucose[10]	X		X	X	X	X		X	
PSA[11]		X		X	X	X		X	X
Serum testosterone, hormones		X[12]		X[12]	X[12]		X	X[12]	
**Tumour Assessments**									
CT/MRI[13]	X				X			X	X[17]
Bone Scan[14]	X[15]				X[15, 14]			X[15]	X[17]
Disease progression Assessments					X		X	X	X
Next therapy for prostate cancer									X

1 If patients continue on study without disease progression or discontinuation of treatment beyond Cycle 12 they should continue visit assessment as follows:

a. Every cycle patients should receive the same assessments as indicated for Cycle 2, Day 1.

b. Tumour assessments and hormone analyses will continue every 3rd cycle (i.e. Cycle 13, 16, 19, 22…)

2 Treatment Discontinuation Visit (TDV) can occur at any scheduled or unscheduled visit when applicable. TDV is meant the day the decision not to continue the treatment is made. At this visit, documentation to confirm progressive disease or unequivocal clinical progression (see 8.3.5) is required. Patients who discontinue treatment due to unacceptable toxicity will be followed until evidence of radiographic progression.

3 End of Treatment Visit should be scheduled to collect safety assessments between 15 to 29 days after the patient stops treatment (TDV).

4 Written informed consent must be obtained within 30 days prior to Cycle 1 Day 1.

5 Weight will be recorded at every visit. Height will be measured at Screening Visit only. Vitals include upright blood pressure, heart rate, and oral or aural body temperature.

6 An ECG and a cardiac ECHO must be obtained at baseline. After 3 months of treatment and thereafter every 3 months, an ECG and ECHO should be repeated if clinically indicated, i.e. in patients with known or suspicion of congestive heart failure (heart failure, uncontrolled hypertension, history of myocardial infarction) or symptoms and signs of heart failure. ECGs should not be obtained when serum potassium is < 3.5 mmol/L. Hypokalemia should be corrected prior to ECG collection.

7 Pre-Treatment SAEs should be reported from time patient signs a consent form up to Day 1 treatment administration.

8 Adverse event follow-up is required for 30 days following last dose to determine if any new or ongoing drug related AE or any SAE regardless of relationship to study drug exists. Follow-up could be conducted by sites via telephone attempts and must be documented in source notes.

9 Liver function tests include: ALK-P, ALT (SGPT), AST (SGOT), GGT, LDH, and total bilirubin and should be conducted in accordance with the current prescribing information (Fachinformation).

10 Glucose can be done as part of Chemistry Panel run by local laboratory. In patients with known diabetes the patient should be advised to measure blood glucose more frequently.

11 If patient undergoes a digital rectal exam (DRE), PSA must be sampled prior to the DRE.

12 Blood specimen for hormone analysis will only be taken at Cycle 1-3 Day 1, at any time of tumour assessment including Treatment Discontinuation visit, and at End-of-Treatment visit.

13 Scans (CT, MRI, and Bone) performed up to 28 days prior to Study Day 1 can be used for baseline assessments. If a status of partial or complete response is made, changes in tumour measurements must be confirmed by repeat assessments that should be performed no less than 4 weeks after the criteria for response are first met.

14 If disease progression is observed on the bone scan, a confirmatory bone scan is required at least 6 weeks later. Study treatment should be continued in the interim unless there is unequivocal clinical progression as defined in Section 7.6. If the confirmatory scan is negative (does not confirm PD), then the patient should be seen again at the next scheduled study visit as specified in the protocol.

15 Bone scan index (BSI) will be analyzed on baseline and at 3 and 6 month after initiation of therapy. For BSI analysis, digitalized bone scans results will be collected and analyzed centrally.

16 Follow-up for all patients ends when rPFS at month 12 can be measured for the last patient on study treatment.

17 For patients who discontinue treatment for reasons other than radiographic progression tumour assessments should continue until confirmed radiographic progression

18 If the Treatment Discontinuation Visit (TDV) occurs ≥21 days after the last scheduled visit, procedures of the next scheduled visit should be done.

Appendix 2 **Study Treatment Preparation and Dispensing Instructions**

Labeling/Packaging

Study treatment (abiraterone acetate) will be provided to each site packaged for patient assignment at the time of randomization. Packaging includes a 30-day supply. Site designated, medically trained personnel will dispense the study treatment to each patient in accordance with this protocol under the guidelines of the site's dispensation standard operating procedure.

Storage of Study Treatments

The study treatment must be stored in a secure area and administered only to patients who entered into the clinical study, at no cost to the patient, in accordance with the conditions specified in this protocol.

Investigational product should be stored at the study site at a room temperature between 15°C to 30°C. Bottles of study drug should be stored with the cap on tightly and should not be refrigerated as this is a high relative humidity environment.

Clinical Supply Inventory

The investigator must keep an accurate accounting of the number of investigational units received from B&C, dispensed to the patients, the number of units returned to the investigator by the patient, and the number of units returned to B&C during and at the completion of the study. A detailed inventory must be completed for the study treatment. The study treatment must be dispensed only by an appropriately qualified person to patients in the study. The treatment is to be used in accordance with the protocol by patients who are under the direct supervision of an investigator.

Return or Disposal of Study Medications/Treatments

All clinical study treatments may be destroyed at the site as specified in writing by Janssen-Cilag GmbH. Study treatments may only be destroyed on written permission of the monitor to ensure monitoring of drug accountability.

Appendix 3 **Protocol-Specific PCWG2 Criteria**

Progressive Disease after Androgen Deprivation Eligibility Criteria (according to PCWG2 [29]):

The reference value (#1) is the last PSA measured before increases are documented, with subsequent values obtained a minimum of 1 week apart. If the PSA at time point 3 (value #3A) is greater than that at point 2, then eligibility has been met. If the PSA is not greater than point 2 (value #3B), but value #4 is, the patient is eligible assuming that other criteria are met, if values 3A or #4 are 2ng/ml or higher.

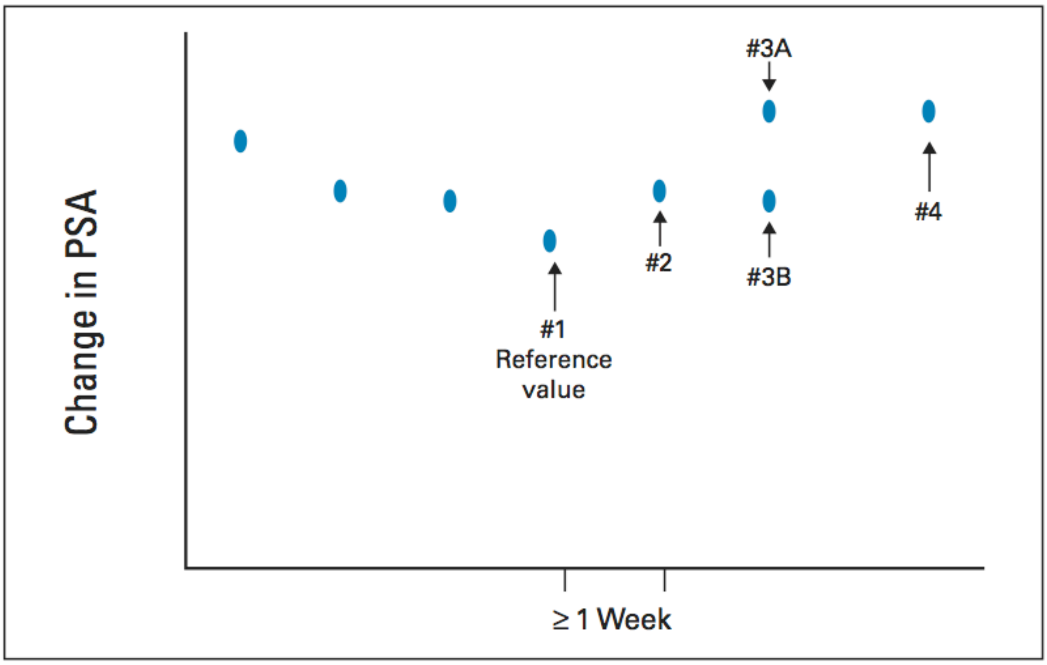

Figure 15.1Eligibility based on PSA

Appendix 4 **Modified Response Evaluation Criteria in Solid Tumours (RECIST)**

Quick Reference for the SPARE-001 study: a randomized phase-II trial of abiraterone acetate plus LHRH-therapy versus abiraterone acetate sparing LHRH-therapy in patients with progressive chemotherapy-naïve castration-resistant prostate cancer (SPARE)

Eligibility

- RECIST criteria will be used on measurable soft tissue and visceral lesions
- Measurable disease - the presence of at least one measurable lesion. If the measurable disease is restricted to a solitary lesion, its neoplastic nature should be confirmed by cytology/histology.
- Measurable lesions – Visceral or extranodal lesions need to be at least ≥10 mm in one dimension using spiral CT; however, lymph nodes need to be ≥20 mm in at least one dimension to be considered as target or evaluable lesions to assess changes in size.
- Non-measurable lesions - all other lesions, including small lesions (longest diameter <20mm), i.e., leptomeningeal disease, ascites, pleural/pericardial effusion, inflammatory breast disease, lymphangitis cutis/pulmonis, cystic lesions, and also abdominal masses that are not confirmed and followed by imaging techniques.
- For this study, bone lesions are not considered "non-measurable" lesions for RECIST. Bone lesions will be assessed by bone scan only.
- All measurements should be taken and recorded in metric notation, using a ruler or calipers. All baseline evaluations should be performed as closely as possible to the beginning of treatment and never more than 28 days before the beginning of the treatment; nodal and visceral/extra-nodal disease will be recorded separately.
- The same method of assessment and the same technique should be used to characterize each identified and reported lesion at baseline and during follow-up.
- Clinical lesions will only be considered measurable when they are superficial (e.g., skin nodules and palpable lymph nodes). For the case of skin lesions, documentation by color photography, including a ruler to estimate the size of the lesion, is recommended.

Methods of Measurement

- CT and MRI are the best currently available and reproducible methods to measure target lesions selected for response assessment. Conventional CT and MRI should be performed with cuts of 10 mm or less in slice thickness contiguously. Spiral CT should be performed using a≤5 mm contiguous reconstruction algorithm.

- Lesions on chest X-ray are acceptable as measurable lesions when they are clearly defined and surrounded by aerated lung. However, CT is preferable.

- Ultrasound (US), endoscopy and laparoscopy should not be used to measure tumour lesions.

- Cytology and histology can be used to differentiate between PR and CR in rare cases (e.g., after treatment to differentiate between residual benign lesions and residual malignant lesions.

Baseline Documentation of "Target" and "Non-Target" Lesions

- All measurable lesions up to a maximum of 5 lesions per organ and 10 lesions in total, representative of all involved organs should be identified as ***target lesions*** and recorded and measured at baseline.

- Target lesions should be selected on the basis of their size (lesions with the longest diameter) and their suitability for accurate repeated measurements (either by imaging techniques or clinically).

- A sum of the longest diameter (LD) for ***all target lesions*** will be calculated and reported as the baseline sum LD. The baseline sum LD will be used as reference by which to characterize the objective tumour.

- All other lesions (or sites of disease) should be identified as ***non-target lesions*** and should also be recorded at baseline. Measurements of these lesions are not required, but the presence or absence of each should be noted throughout follow-up.

Evaluation of Target Lesions

* Complete Response (CR):	Disappearance of all target lesions
* Partial Response (PR):	At least a 30% decrease in the sum of the LD of target lesions, taking as reference the baseline sum LD
* Progressive Disease (PD):	At least a 20% increase in the sum of the LD of target lesions, taking as reference the smallest sum LD recorded since the treatment started or the appearance of one or more new lesions
* Stable Disease (SD):	Neither sufficient shrinkage to qualify for PR nor sufficient increase to qualify for PD, taking as reference the smallest sum LD since the treatment started

Evaluation of Non-Target Lesions

* Complete Response (CR):	Disappearance of all non-target lesions and normalization of tumor marker level
* Incomplete Response/ Stable Disease (SD):	Persistence of one or more non-target lesion(s) or/and maintenance of tumor marker level above the normal limits
* Progressive Disease (PD):	Appearance of new lesions and/or unequivocal progression of existing non-target lesions (1)

(1) Although a clear progression of "non target" lesions only is exceptional, in such circumstances, the opinion of the treating physician should prevail.

Evaluation of Best Overall Response

The best overall response is the best response recorded from the start of the treatment until disease progression/recurrence (taking as reference for PD the smallest measurements recorded since the treatment started). In general, the patient's best response assignment will depend on the achievement of both measurement and confirmation criteria

Response Criteria

Target lesions	Non-Target lesions	New Lesions	Overall Response
CR	CR	No	CR
CR	Incomplete response/SD	No	PR
PR	Non-PD	No	PR
SD	Non-PD	No	SD
PD	Any	Yes or No	PD
Any	PD	Yes or No	PD
Any	Any	Yes	PD

- Patients with a global deterioration of health status requiring discontinuation of treatment without objective evidence of disease progression at that time should be classified as having "symptomatic deterioration". Every effort should be made to document the objective progression even after discontinuation of treatment.
- In some circumstances it may be difficult to distinguish residual disease from normal tissue. When the evaluation of complete response depends on this determination, it is recommended that the residual lesion be investigated (fine needle aspirate/biopsy) to confirm the complete response status.

Reporting of Results

- All patients included in the study must be assessed for response to treatment, even if there are major protocol treatment deviations or if they are ineligible. Each patient will be assigned one of the following categories: 1) complete response, 2) partial response, 3) stable disease, 4) progressive disease, 5) early death from malignant disease, 6) early death from toxicity, 7) early death because of other cause, or 9) unknown (not assessable, insufficient data).

- All of the patients who met the eligibility criteria should be included in the main analysis of the response rate. Patients in response categories 4-9 should be considered as failing to respond to treatment (disease progression). Thus, an incorrect treatment schedule or drug administration does not result in exclusion from the analysis of the response rate. Precise definitions for categories 4-9 will be protocol specific.

- All conclusions should be based on all eligible patients.

Appendix 5 **National Cancer Institute Common Terminology Criteria for AEs**

V4.0 (CTCAE): publish date May 28, 2009:

http://evs.nci.nih.gov/ftp1/CTCAE/CTCAE_4.03_2010-06-14_QuickReference_5x7.pdf

Appendix 6 **ECOG Performance Status**

ECOG Grade Scale (with Karnofsky conversion)

0 Fully active, able to carry on all pre-disease performance without restriction. (Karnofsky 90-100)

1 Restricted in physically strenuous activity but ambulatory and able to carry out work on a light or sedentary nature, eg., light housework, office work. (Karnofsky 70-80)

2 Ambulatory and capable of all self-care but unable to carry out any work activities. Up and about more than 50% of waking hours. (Karnofsky 50-60)

3 Capable of only limited self-care; confined to bed or chair more than 50% of waking hours. (Karnofsky 30-40)

4 Completely disabled. Cannot carry on any self-care. Totally confined to bed or chair. (Karnofsky 10-20)

5 Dead

Appendix 7 **Creatinine Clearance**

Creatinine clearance (CrCl) is to be estimated by using the following formula:

Adult male CrCl = (140 - age) x weight measured in kg
(72 x serum creatinine measured in mg/deciliter)

Appendix 8 **Worst Pain within last 24 hours**

Please rate your pain by marking the box beside the number that best describes your paint at its worst in the last 24 hours

□0 □1 □2 □3 □4 □5 □6 □7 □8 □9 □10

No<br>Pain	Pain as Bad As<br>You can imagine

Appendix 9 **Protocol Definition of Chronic Administration of Opiate Analgesia**

The ‘chronic administration of opiate analgesia’ will be defined as parenteral opiate use for ≥7 days or use of WHO Analgesic Ladder Level 3 oral opiates/opioids for moderate or severe pain for ≥3 weeks (http://www.who.int/cancer/palliative/painladder/en/index.html).

This includes:

Alfentanil

Codeine Phosphate

Codeine Sulfate

Fentanyl

Hydrocodone

Bitartrate

Hydromorphone

Hydromorphone hydrochloride

Levomethadyl Acetate

Levorphanol Tartrate

Meperdine

Methadone

Morphine Sulfate

Oxycodone

Oxymorphone

Propoxyphene

Sufentanil Citrate

Tramadol

Appendix 10 **Sponsor Signatures**

Study Title:	Randomized phase-II trial of abiraterone acetate plus LHRH-therapy versus abiraterone acetate sparing LHRH-therapy in patients with progressive chemotherapy-naïve castration-resistant prostate cancer (SPARE)
Study Number:	SPARE-001/AP 67/11
EudraCT No:	2012-005717-39
Protocoll Date:	
Version Number:	

This clinical study protocol was subject to critical review and has been approved by the sponsor. The following personnel contributed to writing and/or approving this protocol:

Signed:____ Date:____________________

Carsten-Henning Ohlmann, MD

Saarland University

Dept. of Urology

Appendix 11 **Investigator Signatures**

Study Title:	Randomized phase-II trial of abiraterone acetate plus LHRH-therapy versus abiraterone acetate sparing LHRH-therapy in patients with progressive chemotherapy-naïve castration-resistant prostate cancer (SPARE)
Study Number:	SPARE-001/AP 67/11
EudraCT No:	2012-005717-39
Protocoll Date:	
Version Number:	

This clinical study protocol was subject to critical review and has been approved by the sponsor. The following personnel contributed to writing and/or approving this protocol:

Signed:____ Date:____________________

Name of lead investigator at site
site

Signed:____ Date:____________________

Name of co-investigator at site
site
